# Comparative analysis of the rhizosphere microbiome and transcriptome in clubroot-susceptible and resistant rapeseed (*Brassica napus*)

**DOI:** 10.3389/fpls.2026.1729220

**Published:** 2026-04-21

**Authors:** Jingjing Liao, Yuanyuan Cui, Yifan Wang, Xin Zeng, Tingting Chen, Yu Xiang, Diandong Wang

**Affiliations:** School of Advanced Agriculture and Bioengineering, Yangtze Normal University, Chongqing, China

**Keywords:** clubroot, *Plasmodiophora brassicae*, rapeseed, rhizosphere microorganisms, transcriptome

## Abstract

Clubroot disease, caused by *Plasmodiophora brassica*, severely threatens the rapeseed industry in China, with an annual affected area exceeding 667000 hectares. To elucidate the mechanisms in clubroot resistance, we compared the differences in soil physicochemical properties, rhizosphere microbiome, and transcriptomic responses between a susceptible variety, HYZ62 (disease index 54.86), and a resistant variety, HYZ5R (disease index 17.05), following *P. brassicae* infection. The results showed that the electrical conductivity of HYZ5R (R) was 1.73 and 1.57 times that of HYZ62 (S) in the inoculated and uninoculated treatments, respectively. Compared to the 17.18% decrease in alkali-hydrolysable nitrogen content in HYZ62 (S) after inoculation, its content in HYZ5R (R) showed no significant difference. The rhizosphere microbial community significantly differed between HYZ5R (R) and HYZ62 (S), with HYZ5R (R) exhibiting higher relative abundances of several microbial genera, such as *Burkholderia-Caballeronia-Paraburkholderia*, *Humibacter*, *Dyella*, and *Trichoderma*. Although *Bacillus* had a significantly higher relative abundance in the rhizosphere of uninoculated HYZ62 (S), its relative abundance decreased by 30.36% after infection. Transcriptome analysis revealed that, compared to HYZ62 (S), the expression of pattern-triggered immunity-related genes, such as *CML*, *WRKY*, and *PR1*, was higher in HYZ5R (R) and was more strongly induced upon inoculation. Effector-triggered immunity-related genes, such as *RIN4*, *RPS5*, and *HSP90*, were consistently expressed at higher levels. In contrast, HYZ62 (S) showed a broad suppression of defense-related gene expression after inoculation. Furthermore, although *P. brassicae* infection generally suppressed defense-related secondary metabolic pathways, including phenylpropanoid biosynthesis, the expression levels of multiple genes in this pathway remained higher in HYZ5R (R). Together, these results suggest that the higher relative abundances of specific microbial taxa in the rhizosphere and the high expression of defense-related genes are associated with the clubroot resistance in HYZ5R (R).

## 1 Introduction

Rapeseed (Brassicaceae, *Brassica* genus) is a significant oil crop in China. It provides approximately 5.20 million tons of high-quality edible oil annually, accounting for about 47% of the domestic vegetable oil production, and thus plays a crucial role in ensuring edible oil supply (Liu et al., 2019). From 2019 to 2023, rapeseed sowing area increased from 6.583 to 7.805 million hectares, and production rose from 13.485 to 16.317 million tons, averaging 7.094 million hectares and 14.82 million tons per year, respectively. By 2023, rapeseed accounted for 56.06% of the total oil crop sowing area and 42.23% of the total production (National Bureau of Statistics, 2024). However, rapeseed production is severely impacted by clubroot disease, caused by the soil-borne pathogen *Plasmodiophora brassicae*, which infects roots, causes galls that impair water and nutrient uptake, and stunts plant growth (Dixon, 2009). In China, clubroot affects 3.2 to 4.0 million hectares of cruciferous crops annually (about 1/3 of the total cruciferous planting area), causing approximately 20-30% yield losses (Chai et al., 2014). For rapeseed, clubroot affects over 667000 hectares, mainly in Sichuan, Hubei, Hunan, Yunnan, and Anhui provinces, making it the primary threat to rapeseed production safety (Wang et al., 2021).

Disease-resistant varieties are the safest and most effective means against clubroot disease. Multiple dominant loci and QTLs (quantitative trait loci) have been identified on the A and C genomes of *B. napus* (Neik et al., 2017), such as the dominant resistance loci *CRb* and *PbBa8.1* (Shah et al., 2020), *Pb-Bn1* (Manzanares-Dauleux et al., 2000), and the QTLs *Rcr9* and *Rcr10* (Yu et al., 2022), *MCR-A4* and *SCR-C4a* (Li et al., 2016). China’s first conventional *B. napus* variety resistant to clubroot disease, “Huangshuang 5R”, was developed through hybridization between the resistant *B. rapa* variety ECD04 and the susceptible *B. napus* variety Huashuang No. 5, integrating the *PbBa8.1* resistance locus into its genome (Wu et al., 2025a). Meanwhile, the clubroot-resistant *B. rapa* variety Shinki was hybridized with Bing409 to obtain Bing409R, then a new clubroot-resistant hybrid, “Huayouza 62R”, was developed by crossing the male sterile line of “Huayouza 62” with Bing409R, with the *CRb* locus playing a crucial role in conferring resistance (Li et al., 2021). Using “Huashuang 5R” and “Huayouza 62R”, or their parental lines, as resistance sources, China has further cultivated varieties such as “Huayouza 5R”, “Huayouza 160R”, “Fangyou 135R” and “Huayouza 19R” so on (Wu et al., 2025a; Wang et al., 2021).

The function of disease-resistance genes relies on the complex defense program initiated by the host after pathogen infection, involving multiple pathways and various metabolites. Transcriptome sequencing enables the acquisition of gene expression information in different tissues and at different time points during infection. Comparative analysis can elucidate the molecular mechanism and regulatory network underlying plant responses to clubroot infection. For example, Zhang et al. (2025) compared the transcriptome data of CRBJN3–2 plants at 8, 23, and 37 days post-inoculation with different pathotype strains Pb1 and Pb4. Their analysis revealed the differences in hormone signaling and metabolism pathways in the host-*P. brassicae* interaction. Irani et al. (2018) and Ciaghi et al. (2019) explored transcriptomic differences between roots and shoots (compared with uninoculation control), and symptomless roots and galls within the same plant under infection, respectively. They found that genes involved in cell wall modification and JA (jasmonic acid) biosynthesis were up-regulated in infected roots and shoots, and galls. Ciaghi et al. (2019) emphasized that symptomless roots possess a resistance-like transcriptome. Studies by Mei et al. (2019); Wei et al. (2021), and Zhou et al. (2020) elucidated the roles of early signal recognition, ROS (reactive oxygen species), plant hormones, as well as key transcription factors and metabolic pathways in disease resistance through comparisons of resistant and susceptible varieties of *B. napus* and *B. rapa*.

However, plant disease resistance is not solely determined by its own genetic background; the diversity and community of rhizosphere microorganisms, influenced by plant genotypes and soil properties, can also affect plant disease resistance (Xi et al., 2023). In clubroot disease, Murakami et al. (2000), by comparing low-humic and haplic andosols, found that the suppressiveness of soils results from the combined action of both biotic and abiotic factors. Saraiva et al. (2020) further discovered that soils with lower clubroot severity have higher pH, calcium, and magnesium contents, with bacterial communities significantly enriched in beneficial phyla such as Proteobacteria and Acidobacteria, and disease suppressive genera such as *Kaistobacter*, *Bacillus*, and *Bradyrhizobium*. Kang et al. (2024) also confirmed the characteristics of higher pH and calcium content in suppressive soils, along with higher fungal diversity, bacterial abundance, richer beneficial microbial communities, and more complex fungal networks. Daval et al. (2020) compared the clubroot severity of resistant and susceptible rapeseed varieties (Yudal and Tenor) in soils with high, medium, and low microbial diversity. They demonstrated that soil microorganisms affect the plant transcriptome, and that changes in microbial diversity lead to differential expression of a large number of plant defense genes. They also suggested that plant genotype may influence the recruitment process of rhizosphere microorganisms.

Therefore, the resistance of rapeseed to clubroot disease depends not only on resistance genes and corresponding molecular regulatory networks but also on the synergistic interactions between rhizosphere microbial communities and soil physicochemical properties. This study aims to compare differences in soil physicochemical properties and rhizosphere microbial community between clubroot-resistant and susceptible rapeseed varieties before and after inoculation with *P. brassicae*, thereby analyzing the association between varietal resistance and the rhizospheric microenvironment. Simultaneously, through transcriptome analysis, this study seeks to elucidate gene expression differences between the two varieties under inoculated and uninoculated conditions, clarifying the key signaling pathways and regulatory network variations in the response to clubroot infection.

## 2 Materials and methods

### 2.1 Experimental design

**Figure 1:**
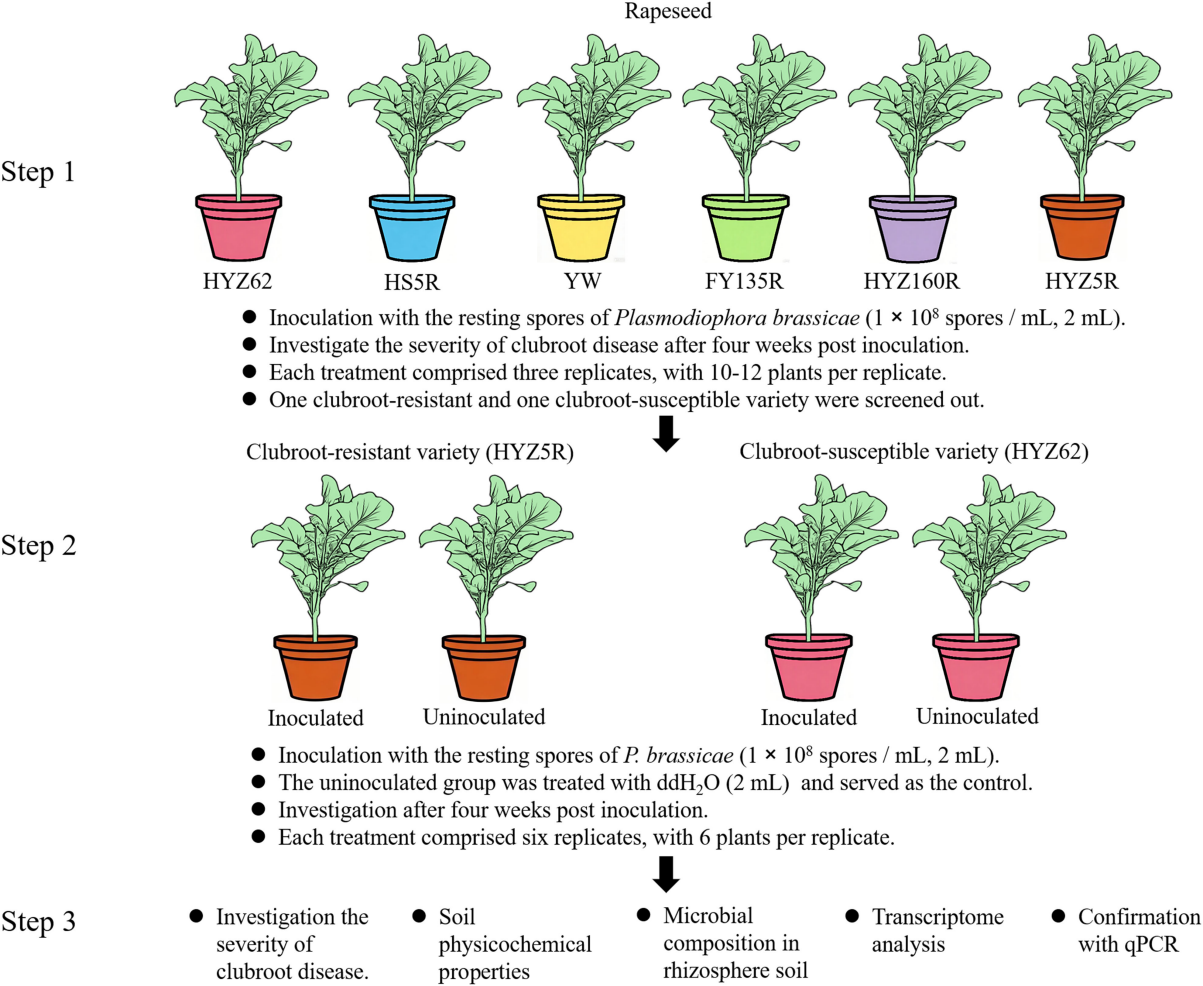
Schematic diagram of the experimental design. HYZ62, HS5R, YW, FY135R, HYZ160R, and HYZ5R were six rapeseed varieties used in this study. Each variety was identified by a uniquely colored pot: HYZ62 (pink), HS5R (blue), YW (yellow), FY135R (green), HYZ160R (purple), HYZ5R (brown). Details of each variety were provided in **Supplementary Table S1**.

Six rapeseed varieties (**Supplementary Table S1**) were used as experimental materials. Through preliminary screening, one clubroot-resistant and one clubroot-susceptible variety were identified. Subsequently, soil physicochemical properties and the microbial composition of rhizosphere soil from these two varieties were analyzed. Combined with transcriptome data, the potential mechanisms underlying the differences in clubroot resistance between these two varieties were elucidated (**Figure 1**).

### 2.2 Seed treatment, seedling transplanting, inoculation, and investigation

Rapeseed seeds were surface-sterilized with 50% 84 disinfectant solution and 75% ethanol, rinsed thoroughly with sterile water, and then sown in seedling pots (9 cm length × 9 cm width × 11 cm height). A pre-mixed and autoclaved substrate, consisting of a 1:3 volume ratio of peat soil to vermiculite, was used as the seedling cultivation medium. The plants were cultivated at 24 °C under a 16/8h light/dark cycle, with continuous moisture maintenance. After approximately one week, when the seedlings had fully expanded cotyledons, they were transplanted individually into pots containing the prepared substrate. The plants were fertilized every three days with a 1000-fold diluted solution of Plant-soul 100% Water soluble fertilizer (20-20-20+TE, Shanghai Wintong Ecological Engineering Co., Ltd). Each variety was replicated three times, with 10–12 plants per replicate.

The clubroot galls collected from the tumorous stem mustard field in Erdu Village, Baisheng Town, Fuling district, were stored in a -20 °C freezer. After retrieval, the galls were rinsed with tap water to remove adhering soil, cut into small pieces, and homogenized with sterile water at a 1:1 (w/v) ratio. The homogenate was filtered through eight layers of sterile gauze, and the filtrate was centrifuged at 100 × g for 5 min to remove the sediment. The resulting supernatant was collected as the resting spore suspension. The concentration of the resting spore suspension was observed and calculated under a microscope with a hemocytometer, and was adjusted to 1 × 10^8^ spores/mL with sterile water to prepare the inoculum. Two weeks after transplanting, each rapeseed seedling was inoculated with 2 mL of the inoculum by pouring it into the root zone.

Four weeks after inoculation, clubroot severity was assessed using a 0–4 scale following the method described by Agarwal et al. (2011). The scoring criteria were defined as follows: 0, no symptoms; 1, small and few galls primarily on the fibrous roots; 2, small and few galls mainly on the taproot; 3, medium-sized galls on both the taproot and fibrous roots; 4, large galls or tissue decay. The DSI (disease index) was calculated using the following formula: DSI = ∑ (n_0_ × 0 + n_1_ × 1 + n_2_ × 2 + n_3_ × 3 + n_4_ × 4) × 100/(N × 4). Here, n_0_ to n_4_ represent the number of plants with disease severity scores of 0 to 4, respectively, and N denotes the total number of plants assessed. Based on the DSI value, resistance was classified into three categories: immunity (I) (DSI = 0), resistant (R) (0 < DSI ≤ 20), susceptible (S) (20 < DSI ≤ 100). This classification was slightly modified from the criteria established by Li et al. (2021). DI (disease incidence) was calculated using the following formula: DI = (Number of infected plants/Total number of plants) × 100%.

### 2.3 The sampling of bulk soil, rhizosphere soil, and root tissues of clubroot-resistant and clubroot-susceptible rapeseed varieties

To comprehensively explore the mechanisms of clubroot resistance, we analyzed three key components. First, root tissues, as the primary site of colonization and pathogenesis by *P. brassicae*, were subjected to transcriptome sequencing to elucidate the host’s direct molecular responses (Dixon, 2009; Zhang et al., 2025). Second, rhizosphere soils were collected to profile microbial community, reflecting plant-microbe interactions during disease progression (Xi et al., 2023). Finally, the physicochemical properties of bulk soil reflect the soil environment of the entire system, which is known to influence clubroot occurrence (Bhering et al., 2020).

HYZ5R was selected as the clubroot-resistant variety, and HYZ62 was selected as the clubroot-susceptible variety. Field soil (0 to 20 cm surface soil) was collected from Xiaowan, Zhenxi town, Fuling district, Chongqing (29°57′28″ N and 107°31′31″ E). After natural air-drying, the soil was crushed, thoroughly mixed, and filled into seedling pots (9 cm length × 9 cm width × 11 cm height). The seedlings were transplanted into the pots after full cotyledon expansion, with two seedlings per pot. They were cultivated at 24 °C under a 16/8h light/dark cycle. Four treatments were established: inoculated HYZ62 (Ino_HYZ62), uninoculated HYZ62 (Uni_HYZ62), inoculated HYZ5R (Ino_HYZ5R), and uninoculated HYZ5R (Uni_HYZ5R). Each treatment included six replicates. One biological replicate consisted of three pots (with two seedlings per pot), yielding a total of six plants per replicate. Two weeks after transplanting, each rapeseed seedling was inoculated with 2 mL of resting spore suspension at a concentration of 1 × 10^8^ spores/mL. An equal volume of sterile water was applied as the control.

At four weeks post-inoculation, the rapeseed plants were uprooted, and the bulk soil loosely adhering to the roots was shaken off onto sterilized kraft paper. The bulk soil from six plants per replicate was combined into one composite sample. The roots of these six plants were then immersed in 45 mL of sterile PB (phosphate buffer, pH 7.0, 0.5 mM) and vigorously shaken by hand until no visible soil remained attached to the root surface. This washing process was repeated three times using 45 mL of fresh sterile PB each time, yielding a total of 135 mL of rhizosphere soil suspension. The suspension was centrifuged at 8000 rpm for 5 min, after which the supernatant was discarded. The pellet was flash-frozen in liquid nitrogen and stored at -80 °C. Subsequently, the roots were rinsed with tap water, gently blotted dry, scored for clubroot severity, and photographed. Finally, the roots from all six plants were wrapped in aluminum foil, flash-frozen in liquid nitrogen, and stored at -80 °C.

### 2.4 Determination of soil physicochemical properties

The following six parameters of the physicochemical properties of bulk soil samples were measured by Convinced-Test Company (Nanjing, China) according to the cited national standard methods: EC (electrical conductivity; electrode method, HJ 802-2016), pH (glass electrode method, NY/T 1121.2-2006), SOC (soil organic carbon; potassium dichromate oxidation-external heating method, NY/T 1121.6-2006), AN (alkali-hydrolysable nitrogen; alkaline hydrolysis diffusion method, LY/T 1229-1999), AP (available phosphorus; extracted with ammonium fluoride-hydrochloric acid solution and determined by molybdenum-antimony anti-colorimetric method, NY/T 1121.7-2014), and AK (available potassium; extracted with cold 2 mol/L HNO_3_ solution and determined by flame photometry).

### 2.5 High-throughput sequencing analysis of rhizosphere soil bacteria and fungi

Rhizosphere soil samples were retrieved from the -80°C freezer, freeze-dried, ground, and homogenized using a sterile mortar and pestle. The processed samples were sent to Shanghai Majorbio Co., Ltd. for high-throughput sequencing analysis. Bacterial and fungal sequences were amplified using the primer pairs 338F/806R and ITS1/ITS2, respectively. The raw data obtained from the MiSeq sequencing platform were processed using fastp and FLASH for sequence optimization. Bases with quality values below 20 at the ends of reads were filtered out. A 10-bp sliding window was applied; if the average quality within the window dropped below 20, the subsequence starting from the beginning of the window was trimmed. Reads shorter than 50 bp after quality control, and those containing ambiguous bases (N), were removed. Paired-end reads were merged into a single sequence based on sequence overlap, with a minimum overlap length of 10 bp. The maximum allowed mismatch ratio in the overlap region was set to 0.2; sequences not meeting this criterion were discarded. Demultiplexing was performed using the barcode and primer sequences at the ends of the reads, with sequence orientation adjusted accordingly. No mismatches were allowed in the barcode region, while up to two mismatches were permitted in the primer sequence.

The optimized sequences were further processed using a denoising approach (DADA2) to minimize PCR amplification and sequencing errors, generating the actual biological sequences represented as ASVs (amplicon sequence variants). Based on the ASV sequences and their abundance information, subsequent analyses were performed, including taxonomic classification, community diversity assessment, and species composition analysis. The databases used for bacterial and fungal taxonomic annotation were Silva138/16s_bacteria and Unite8.0/its_fungi, respectively. Taxonomic assignment was performed with the classify-sklearn method (Naïve Bayes), with a confidence threshold of 0.7.

The data were analyzed on the Majorbio Cloud Platform (www.majorbio.com) (Ren et al., 2022). The α-diversity was evaluated based on ASV-level metrics, including the Sobs index (reflecting community richness), the Shannon index (reflecting community diversity), and the Pielou_e index (reflecting community evenness). For β-diversity analysis, PCoA (Principal Coordinates Analysis) was performed at the ASV-level, with Bray-Curtis distance employed to quantify dissimilarities between samples. Group differences were assessed using ANOSIM (analysis of similarities). Phyla and genera with a relative abundance greater than 1% were selected to generate stacked bar charts illustrating community composition. A heatmap of community composition was produced using the Omicshare platform (https://www.omicshare.com/tools/).

### 2.6 Transcriptome sequencing and qPCR analysis

Root samples from six biological replicates per treatment were randomly pooled into three composite samples (each containing two original replicates), and sent to Chongqing Gene Sci-Tech Co., Ltd. for transcriptome sequencing. The raw image data obtained from DNBSEQ-T7 HiSeq sequencing were converted into sequence data through base calling, yielding raw sequencing data files. The raw data were processed using Trimmomatic software to remove adapters and low-quality reads. Subsequently, a new round of quality control was conducted on the cleaned reads, and metrics including Q20, Q30, and GC content were summarized. The clean data were aligned to the *B. napus* reference genome Darmor-bzh V10 (Brana_Dar_V10) (Rousseau-Gueutin et al., 2020; https://yanglab.hzau.edu.cn/BnIR/germplasm_info?id=Darmor.v10), and the mapping statistics for each sample were calculated. The average sequencing depth across the 12 samples was 5.45 G, with the alignment percentage to the reference genome ranging from 76.65% to 96.75%. The average mapping rate was 90.88%, and on average, 95.55% of the sequenced reads were uniquely aligned to the genome (**Dataset 1**).

Differential gene expression analysis was conducted using edgeR (version 3.42.4). Raw read counts were normalized using the TMM (trimmed mean of M-values) method to correct for library size differences. A negative binomial generalized linear model was fitted to the count data to estimate dispersion parameters and account for biological variability. log_2_FC was calculated from the model coefficients representing the contrast between treatment and control groups. Statistical significance was determined using edgeR’s exact test (for simple comparisons) or quasi-likelihood F-test (for complex designs), which are specifically designed for overdispersed count data. The resulting *P*-values were adjusted for multiple testing using the Benjamini-Hochberg procedure to obtain FDR. DEGs (differentially expressed genes) were screened using the criteria of |log_2_FC| ≥ 1 and an FDR (false discovery rate) < 0.05. Gene function annotation was performed using GO (gene ontology) and KEGG (kyoto encyclopedia of genes and genomes). PCA (principal component analysis) of gene expression and a Venn diagram of DEGs across the four treatments were generated using the Omicshare platform.

To validate the RNA sequencing results, six genes were randomly selected for qPCR (quantitative PCR) validation. Total RNA was extracted from the root samples using RNAiso Plus (Takara, 9109). RNA concentration and purity were assessed by Nanodrop One (Thermo Fisher), and only samples with A260/A280 ratios between 1.8-2.0 were used for subsequent analysis. Genomic DNA was removed using the gDNase mix included in the RT EasyTM II kit (Foregene, RT-01032), followed by reverse transcription to synthesize cDNA. qPCR was performed using this cDNA as a template on the ABI 7900HT system with the SYBR Green master mix (Foregene, QP-01012). The details of the six target genes and the reference gene *BnTUB* (LOC111213844), and primers were provided in **Supplementary Table S2**. All procedures were carried out in accordance with the manufacturer’s instructions. Relative expression was calculated using the 2^-ΔΔCT^ method (Livak and Schmittgen, 2001). Each treatment included three biological replicates, with three technical replicates.

### 2.7 Statistical analysis

IBM SPSS Statistics 22 was utilized for general statistical analysis. The DI and DSI among the six rapeseed varieties were analyzed using one-way ANOVA (analysis of variance), followed by the LSD (least significant difference) *post-hoc* test, with a significance level set at *P* < 0.05. For the comparison between two sets of data (specifically, the same variety with and without inoculation, and resistant versus susceptible varieties), Student’s t-test was applied.

## 3 Results

### 3.1 Assessment of clubroot resistance in six rapeseed varieties

**Figure 2:**
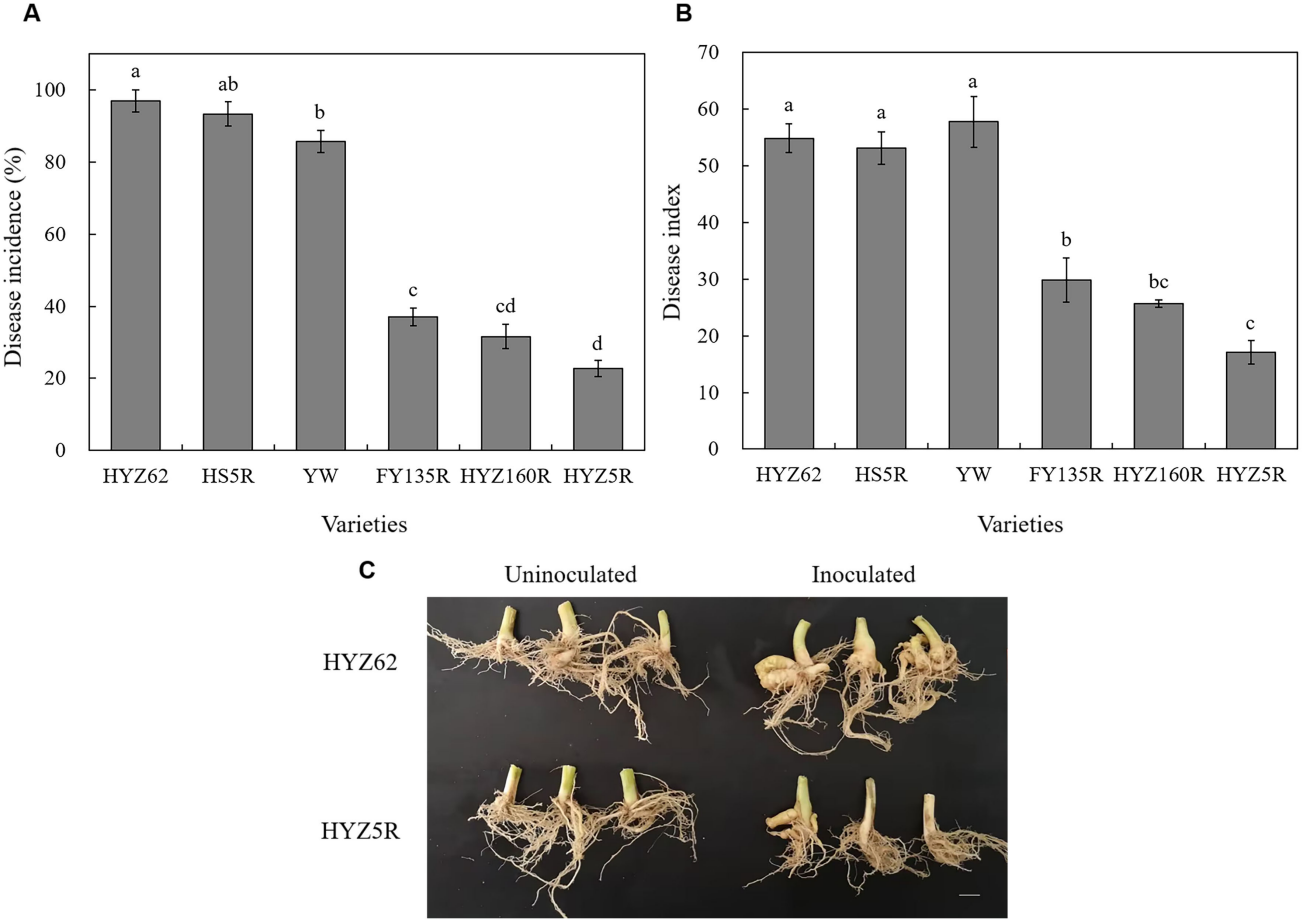
Variation in clubroot severity across six rapeseed varieties. **(A)** Disease incidence (DI, %). **(B)** Disease index (DSI). **(C)** Root phenotypes of HYZ5R (R) and HYZ62 (S). HYZ62, HS5R, YW, FY135R, HYZ160R, HYZ5R were six rapeseed varieties (**Supplementary Table S1**). Values (**Supplementary Table S3**) in **(A)** and **(B)** represent the mean of three biological replicates; error bars represent the standard error (SE). Plants in the Uninoculated and Inoculated treatments were treated with 2 mL of water and resting spore suspension (1 × 10^8^ resting spores/mL), respectively. All data and images were collected at four weeks post-inoculation.

The results (**Figure 2**; **Supplementary Table S3**) showed that among the six varieties, the highest and lowest clubroot disease incidence were found in HYZ62 (96.67%) and HYZ5R (22.73%), respectively, with DSIs of 54.86 and 17.05. Therefore, HYZ62 and HYZ5R were selected as susceptible (S) and resistant (R) varieties, respectively, for subsequent experiments.

### 3.2 Differences in soil physicochemical properties between HYZ62 (S) and HYZ5R (R)

**Figure 3:**
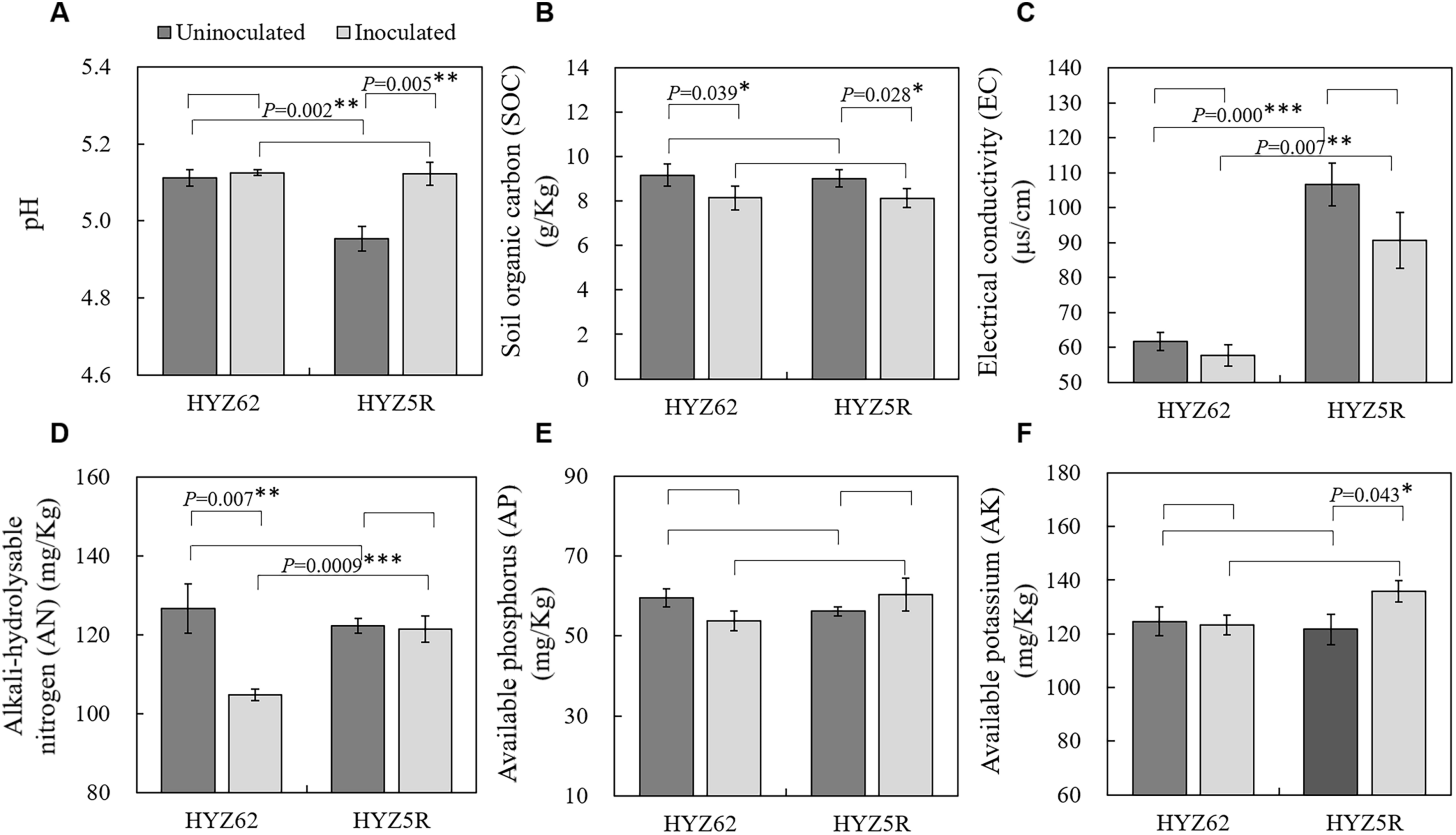
Soil physicochemical properties between HYZ62 (S) and HYZ5R (R). **(A)** pH. **(B)** Soil organic carbon (SOC; g/Kg). **(C)** Electrical conductivity (EC, μs/cm). **(D)** Alkali-hydrolysable nitrogen (AN; mg/Kg). **(E)** Available phosphorus (AP; mg/Kg). **(F)** Available potassium (AK; mg/Kg). Values represent the mean of six biological replicates; error bars represent SE. Asterisks indicate significant differences between groups as determined by the t-test (*P < 0.05, **P < 0.01, ***P < 0.001). HYZ62 and HYZ5R were two rapeseed varieties (**Supplementary Table S1**). Plants in the Uninoculated and Inoculated treatments were treated with 2 mL of water and resting spore suspension (1 × 10^8^ resting spores/mL), respectively. All data were collected at four weeks post-treatment.

The results of soil physicochemical properties for HYZ62 (S) and HYZ5R (R) (**Figure 3**) showed significant changes in soil pH, EC (electrical conductivity), SOC (soil organic carbon), and AN (alkali-hydrolysable nitrogen) content. In uninoculated treatments, the soil pH of HYZ62 (S) was significantly higher than that of HYZ5R (R), but both values fell within the optimal range for clubroot infection. In inoculated treatments, there was no significant difference in soil pH. The soil EC of HYZ5R (R) was significantly higher than that of HYZ62 (S), both under uninoculated and inoculated conditions. Although SOC content decreased significantly after *P. brassica* infection in both varieties, there was no significant difference between them under inoculation. No significant difference was observed in soil AN content between the two varieties in the uninoculated condition. However, in inoculation treatments, the soil AN content of HYZ62 (S) decreased significantly. At the same time, that of HYZ5R (R) remained unchanged, resulting in a considerably lower soil AN content in HYZ62 (S) compared to HYZ5R (R). For AP (available phosphorus) and AK (available potassium), no significant differences were detected.

### 3.3 Differences in rhizosphere soil bacterial and fungal communities between HYZ62 (S) and HYZ5R (R)

#### 3.3.1 Summary of high-throughput sequencing dataset

High-throughput sequencing of 24 rhizosphere soil samples (**Dataset 2**) yielded a total of 3000561 bacterial and 3033242 fungal raw reads. After quality filtering and noise reduction, an average of 39900 bacterial and 66156 fungal reads per sample were retained, corresponding to 1944 bacterial and 296 fungal ASVs. To ensure comparability across samples, the bacterial and fungal datasets were rarefied to an even depth of 28666 and 50423 per sample, respectively. Subsequent clustering of these rarefied sequences yielded an average of 653 and 235 high-quality bacterial and fungal ASVs per sample, respectively.

#### 3.3.2 Alpha-diversity analysis of bacterial and fungal communities in the rhizosphere soil of HYZ62 (S) and HYZ5R (R)

**Figure 4:**
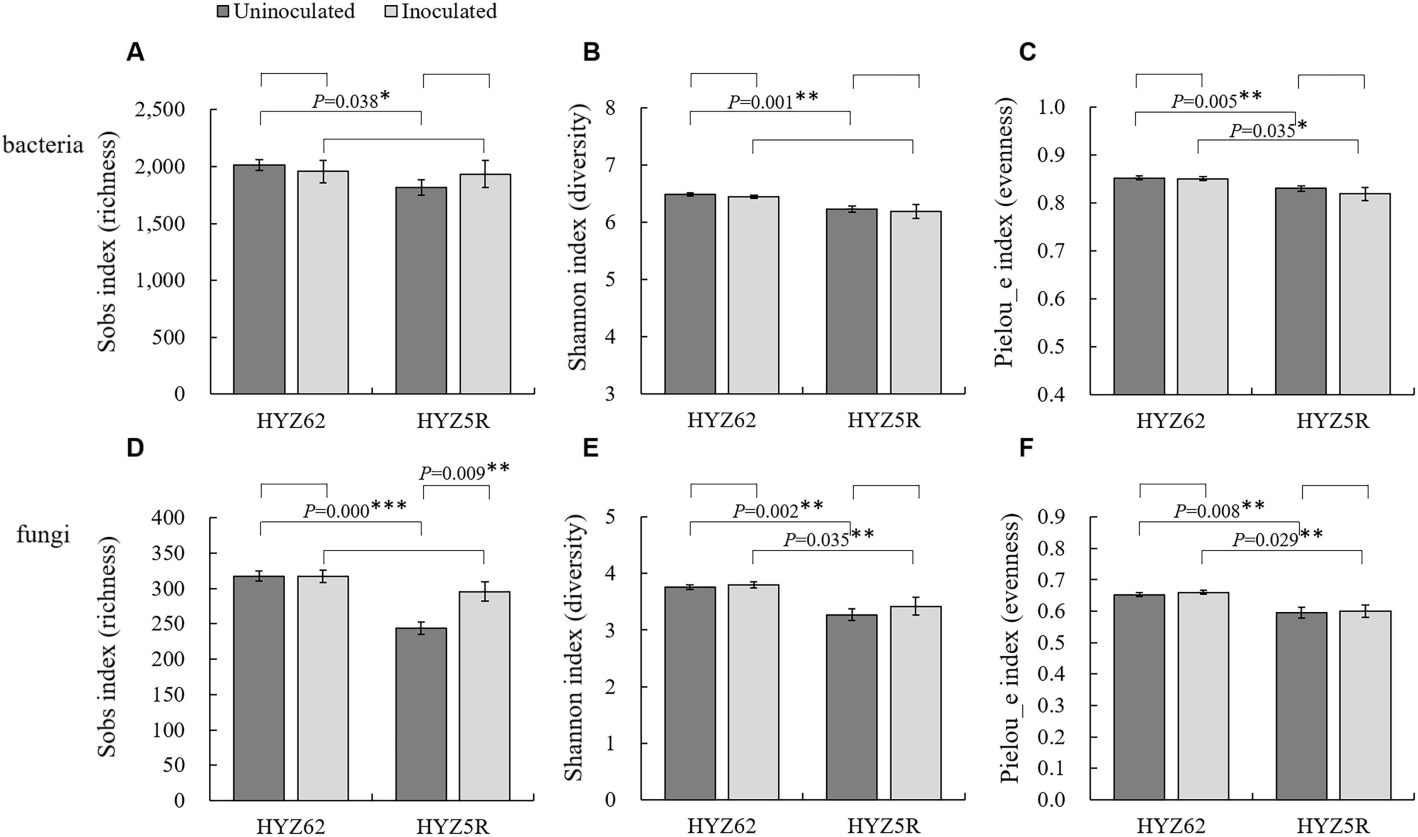
Alpha-diversity of bacterial and fungal communities in HYZ62 (S) and HYZ5R (R). Bacterial and fungal α-diversity indices: Sobs index **(A, D)**, Shannon index **(B, E)**, and Pielou_e index **(C, F)**. Values represent the mean of six biological replicates; error bars represent SE. Asterisks indicate significant differences between groups as determined by the t-test (*P < 0.05, **P < 0.01, ***P < 0.001). HYZ62 and HYZ5R were two rapeseed varieties (**Supplementary Table S1**). Plants in the Uninoculated and Inoculated treatments were treated with 2 mL of water and resting spore suspension (1 × 10^8^ resting spores/mL), respectively. All data were collected at four weeks post-treatment and were analyzed at the ASV-level.

Significant differences in α-diversity indices (Sobs, Shannon, and Pielou_e) were observed between HYZ62 (S) and HYZ5R (R) under the uninoculation condition, with HYZ62 (S) showing higher values across all indices (**Figure 4**). In inoculation treatments, Pielou_e index (Pielou’s evenness, reflecting community evenness) of both bacterial and fungal communities, as well as the fungal Shannon index (reflecting community diversity), remained significantly higher in HYZ62 (S). The Sobs index (observed species, reflecting community richness) increased significantly in HYZ5R (R) under inoculation.

#### 3.3.3 Beta-diversity analysis of bacterial and fungal communities in the rhizosphere soil of HYZ62 (S) and HYZ5R (R)

**Figure 5:**
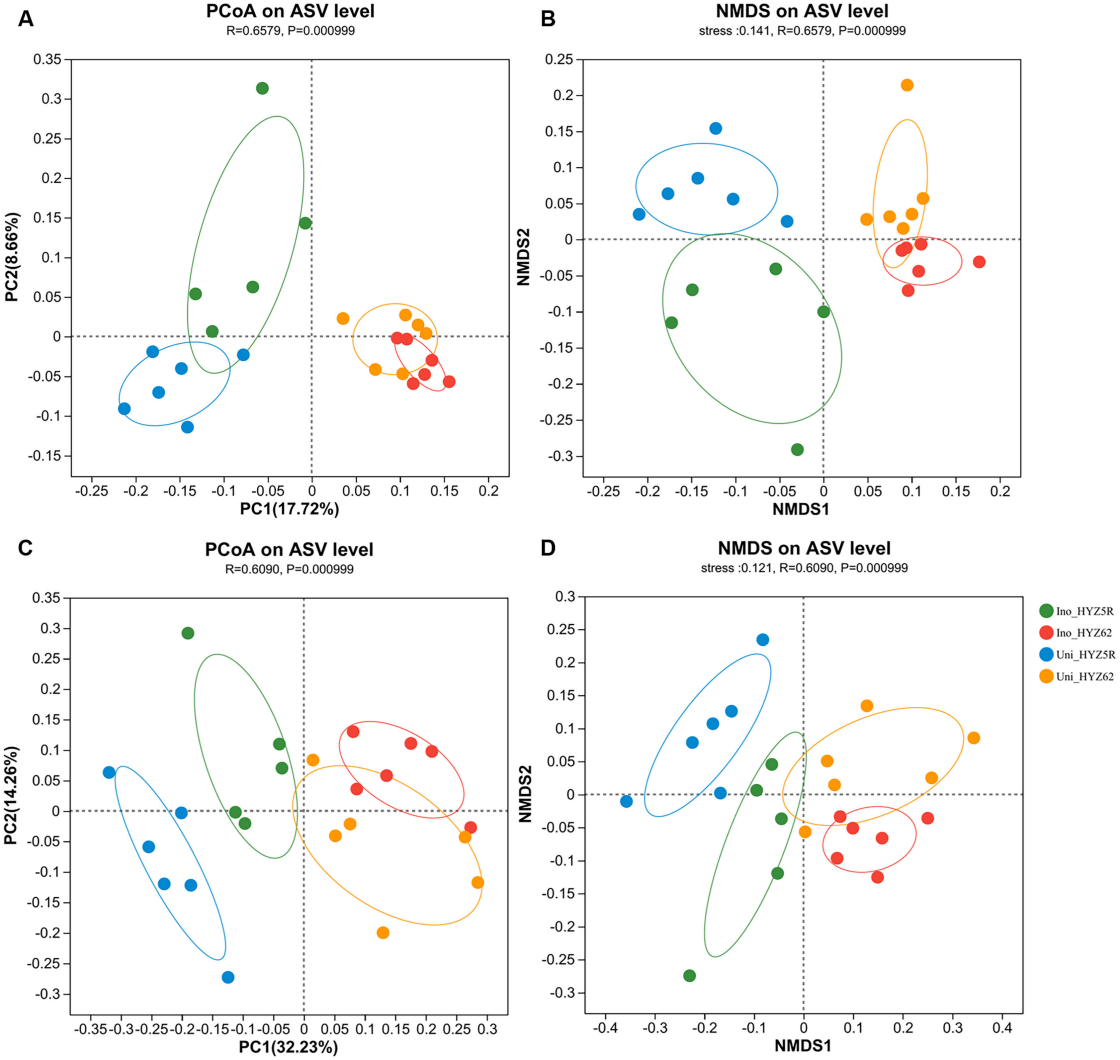
PCoA and NMDS analyses of bacterial and fungal communities in HYZ62 (S) and HYZ5R (R) at the ASV-level. PCoA and NMDS plots for bacteria **(A, B)** and fungi **(C, D)**. The analysis of the community was performed based on ASV-level data using the Bray-Curtis distance. The experimental treatments included: inoculated HYZ62 (Ino_HYZ62), uninoculated HYZ62 (Uni_HYZ62), inoculated HYZ5R (Ino_HYZ5R), and uninoculated HYZ5R (Uni_HYZ5R). HYZ62 and HYZ5R were two rapeseed varieties (**Supplementary Table S1**). Plants in the Uninoculated and Inoculated treatments were treated with 2 mL of water and resting spore suspension (1 × 10^8^ resting spores/mL), respectively.

The results (**Figure 5**) of the PCoA and NMDS based on Bray-Curtis dissimilarity revealed a clear separation of bacterial and fungal communities between HYZ5R (R) and HYZ62 (S) along the PC1 axis. The samples of HYZ5R (R) clustered on the left side of PC1, whereas those of HYZ62 (S) clustered on the right. For bacterial communities, PC1 and PC2 explained 17.72% and 8.66% of the variation, respectively, collectively accounting for 26.38% of the total variance. For fungal communities, PC1 and PC2 explained 32.23% and 14.26% of the variation, respectively, together representing 46.49% of the total variance. Moreover, inoculation induced relatively minor but significant changes in the bacterial community of HYZ62 (S), while causing substantial shifts in its fungal community. In contrast, both the bacterial and fungal communities of HYZ5R (R) were markedly altered by inoculation.

#### 3.3.4 Compositional differences in rhizosphere bacteria and fungi at the phylum and genus levels

**Figure 6:**
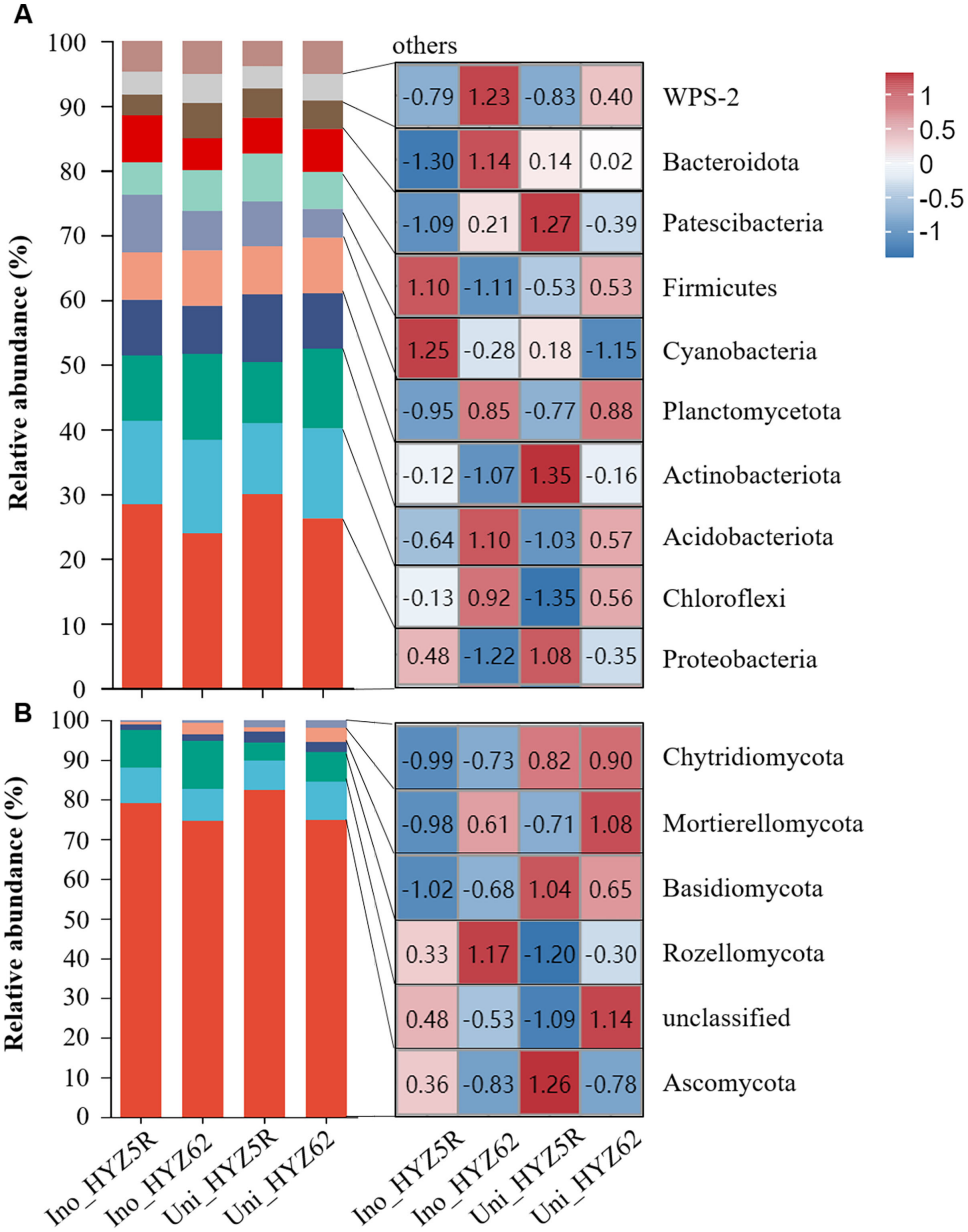
Phylum-level community composition and abundance heatmap of bacteria and fungi. **(A)** Bacterial and **(B)** fungal community. For each, the relative abundance (stacked bar plot, left) and the abundance of dominant phyla (heatmap, right) are displayed together. The experimental treatments included: inoculated HYZ62 (Ino_HYZ62), uninoculated HYZ62 (Uni_HYZ62), inoculated HYZ5R (Ino_HYZ5R), and uninoculated HYZ5R (Uni_HYZ5R). HYZ62 and HYZ5R were two rapeseed varieties (**Supplementary Table S1**). Plants in the Uninoculated and Inoculated treatments were treated with 2 mL of water and resting spore suspension (1 × 10^8^ resting spores/mL), respectively.

The bacterial community was dominated by ten phyla with relative abundances > 1% (collectively 95.30%): Proteobacteria (~27.07%), Chloroflexi (~13.07%), Acidobacteriota (~11.33%), Actinobacteriota (~8.76%), Planctomycetota (~8.01%), Cyanobacteria (~6.49%), Patescibacteria (~6.05%), Firmicutes (~6.16%), Bacteroidota (~4.43%), and WPS-2 (~3.93%) (**Figure 6A**; **Supplementary Table S4**). In uninoculated treatments, higher abundances of Proteobacteria and Cyanobacteria were found in HYZ5R (R), whereas higher abundances of Chloroflexi and Acidobacteriota were detected in HYZ62 (S). The inoculation treatment significantly altered the abundance of three phyla in HYZ5R (R): Chloroflexi increased, while Patescibacteria and Bacteroidota decreased. Consequently, the final composition of the inoculated HYZ5R (R) was characterized by significantly lower relative abundances of five phyla compared to HYZ62 (S): Chloroflexi, Acidobacteriota, Patescibacteria, Bacteroidota, and WPS-2.

The fungal community was dominated by five phyla and one unclassified group with relative abundances > 1% (collectively 91.48%): Ascomycota (~77.65%), Rozellomycota (~8.34%), Basidiomycota (~2.11%), Mortierellomycota (~2.13%), Chytridiomycota (1.25%), and unclassified (~8.51%) (**Figure 6B**; **Supplementary Table S4**). In uninoculated treatments, the community of HYZ5R (R) was characterized by a significantly higher relative abundance of Ascomycota and a significantly lower abundance of Mortierellomycota compared to HYZ62 (S). In inoculated treatments, the difference in Mortierellomycota abundance between HYZ5R (R) and HYZ62 (S) remained statistically significant.

**Figure 7:**
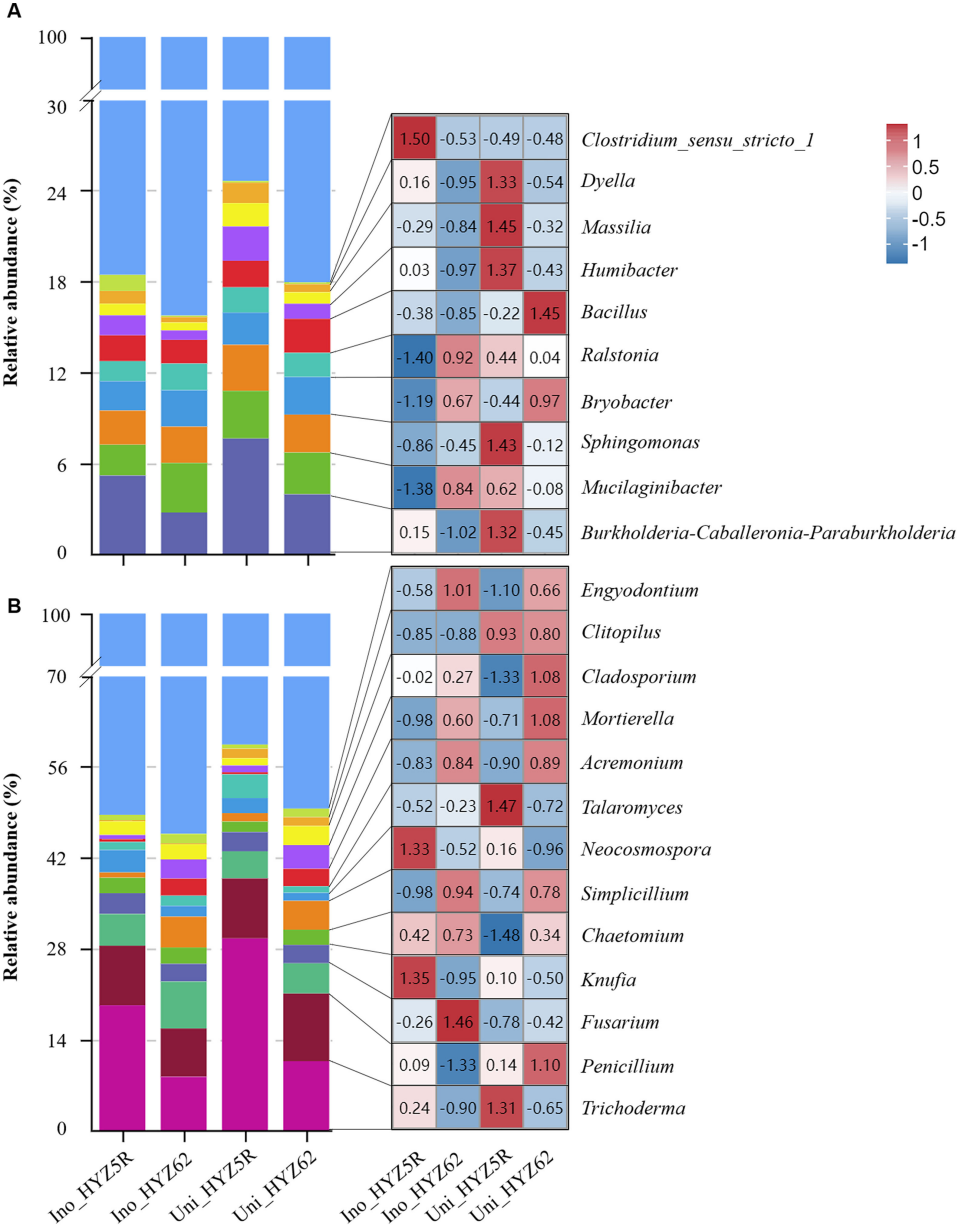
Genus-level community composition and abundance heatmap of bacteria and fungi. **(A)** Bacterial and **(B)** fungal community. For each, the relative abundance (stacked bar plot, left) and the abundance of dominant genera (heatmap, right) are displayed together. The experimental treatments included: inoculated HYZ62 (Ino_HYZ62), uninoculated HYZ62 (Uni_HYZ62), inoculated HYZ5R (Ino_HYZ5R), and uninoculated HYZ5R (Uni_HYZ5R). HYZ62 and HYZ5R were two rapeseed varieties (**Supplementary Table S1**). Plants in the Uninoculated and Inoculated treatments were treated with 2 mL of water and resting spore suspension (1 × 10^8^ resting spores/mL), respectively.

A total of 10 bacterial and 13 fungal genera with relative abundances > 1% were identified (**Figure 7**; **Supplementary Table S5**). Analysis of differential bacterial genus abundance showed that, in uninoculated treatments, the relative abundances of *Sphingomonas*, *Humibacter*, and *Massilia* were significantly higher in HYZ5R (R) than in HYZ62 (S), whereas that of *Bacillus* was significantly lower. In inoculated treatments, the relative abundance of *Humibacter*, *Massilia*, and *Dyella* decreased significantly in both varieties. Additionally, in HYZ5R (R), the abundances of *Burkholderia-Caballeronia-Paraburkholderia*, *Mucilaginibacter*, and *Sphingomonas* also reduced significantly, while a significant decrease was observed for *Bacillus* in HYZ62 (S). Ultimately, the final relative abundances of *Burkholderia-Caballeronia-Paraburkholderia*, *Humibacter*, and *Dyella* remained significantly higher in HYZ5R (R) than in HYZ62 (S). In contrast, the abundances of *Mucilaginibacter* and *Bryobacter* were significantly lower in HYZ5R (R).

Analysis of differential fungal genus abundance showed that, in uninoculated treatments, the relative abundance of *Trichoderma* was significantly higher in HYZ5R (R) than in HYZ62 (S). At the same time, that of *Mortierella* was significantly lower. In inoculated treatments, the relative abundances of *Simplicillium*, *Acremonium*, and *Mortierella* were significantly lower in HYZ5R (R) than in HYZ62 (S).

### 3.4 Comparative transcriptome analysis reveals key gene expression differences between HYZ62 (S) and HYZ5R (R)

**Figure 8:**
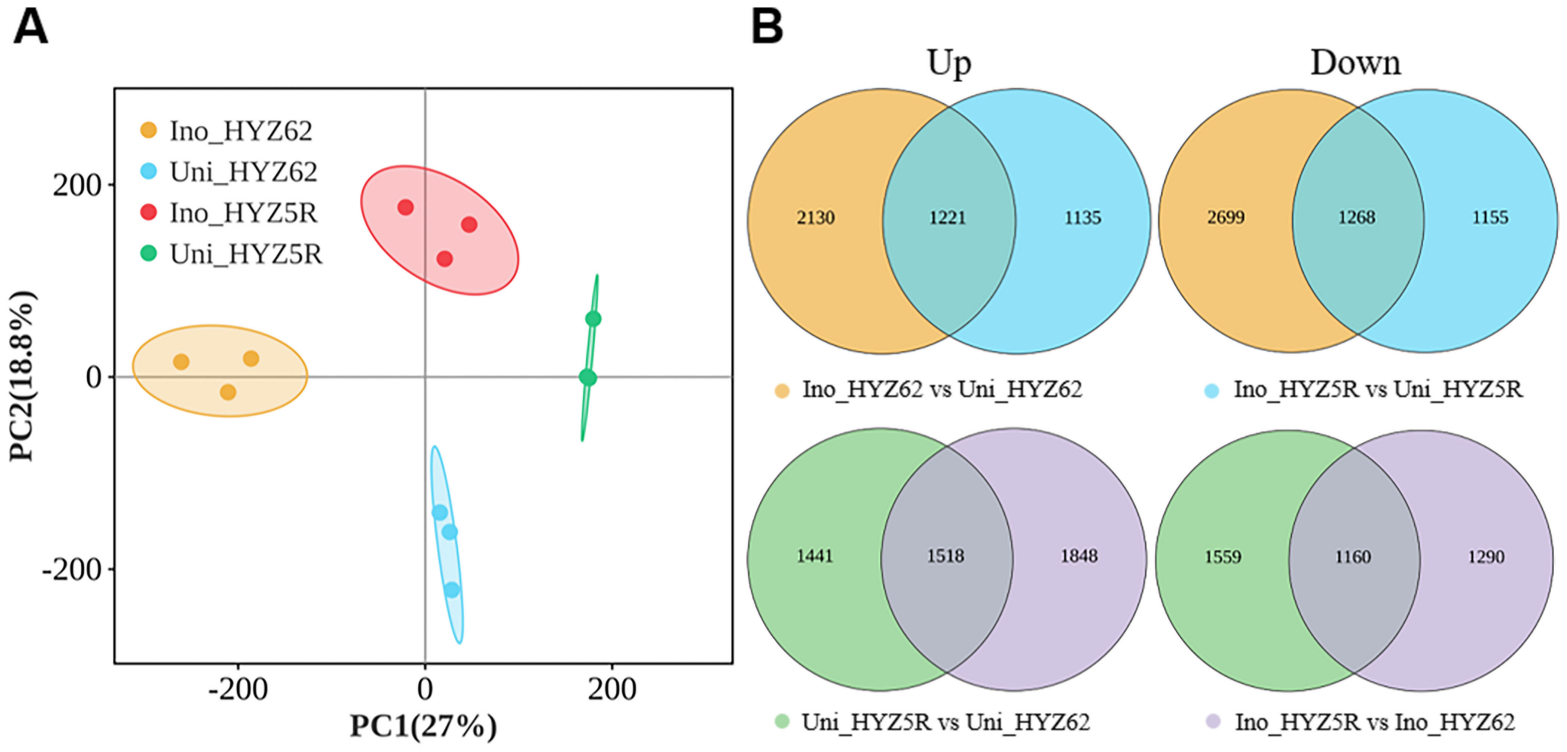
Overview of transcriptomic profiles and differential gene expression. **(A)** PCA of all samples based on gene expression profiles. **(B)** Venn diagrams showing the number of DEGs in pairwise comparisons between different treatments. The experimental treatments included: inoculated HYZ62 (Ino_HYZ62), uninoculated HYZ62 (Uni_HYZ62), inoculated HYZ5R (Ino_HYZ5R), and uninoculated HYZ5R (Uni_HYZ5R). HYZ62 and HYZ5R were two rapeseed varieties (**Supplementary Table S1**). The yellow, blue, green and purple circles correspond to the DEGs from four comparison groups: Ino_HYZ62 vs Uni_HYZ62, Ino_HYZ5R vs Uni_HYZ5R, Uni_HYZ5R vs Uni_HYZ62, and Ino_HYZ5R vs Ino_HYZ62, respectively. Plants in the Uninoculated and Inoculated treatments were treated with 2 mL of water and resting spore suspension (1 × 10^8^ resting spores/mL), respectively.

To investigate the responses of HYZ5R (R) and HYZ62 (S) to *P. brassicae* infection, comparative transcriptome analysis was conducted on both inoculated and uninoculated plants. PCA revealed significant gene expression changes in both varieties (**Figure 8A**). The first two principal components (PC1 and PC2) together explained 45.8% of the total variance among treatments. Differential gene expression analysis (**Figure 8B**) identified a total of 5678 DEGs when comparing Uni_HYZ5R with Uni_HYZ62 (green circles), comprising 2959 (1441 + 1518) up-regulated and 2719 (1559 + 1160) down-regulated genes. The comparison between Ino_HYZ5R and Ino_HYZ62 (purple circles) revealed 5816 DEGs, with 3366 (1518 + 1848) up-regulated and 2450 (1160 + 1290) down-regulated. Among these DEGs, 1518 and 1160 were commonly up-regulated and down-regulated, respectively, in HYZ5R (R) compared to HYZ62 (S), regardless of inoculation status. A comparison between inoculated and uninoculated plants identified 7318 DEGs in HYZ62 (S) (yellow circles), 3351 (2130 + 1221) up-regulated, 3967 (2699 + 1268) down-regulated; and 4779 in HYZ5R (R) (blue circles), 2356 (1221 + 1135) up-regulated, 2423 (1268 + 1155) down-regulated. Among these, 1221 and 1268 genes were commonly up-regulated and down-regulated, respectively, in both varieties upon *P. brassicae* infection. KEGG pathways analysis revealed that the DEGs were significantly enriched in several pathways, including plant-pathogen interaction, plant hormone signal transduction, phenylpropanoid biosynthesis, MAPK signaling pathway, and biosynthesis of secondary metabolites, and so on (**Supplementary Figure S1**). Based on this result, we conducted a detailed examination of the DEGs associated with the plant-pathogen interaction pathway (ko04626) and those involved in the biosynthesis of plant defense-related secondary metabolites.

To validate the transcriptome results, the expression patterns of six selected genes (**Supplementary Table S2**) were examined by qPCR. The bar graph depicts the log_2_FC change from the RNA-seq data, while the line graph shows the relative expression level determined by qPCR (**Supplementary Figure S2**). The expression trends revealed by qPCR for all tested genes were consistent with the transcriptome data. Specifically, genes that were up-regulated or down-regulated in the RNA-seq analysis displayed corresponding increases or decreases in qPCR. This concordance between the two methods confirms the reliability of our transcriptome results.

#### 3.4.1 DEGs in the plant-pathogen interaction pathway

**Figure 9:**
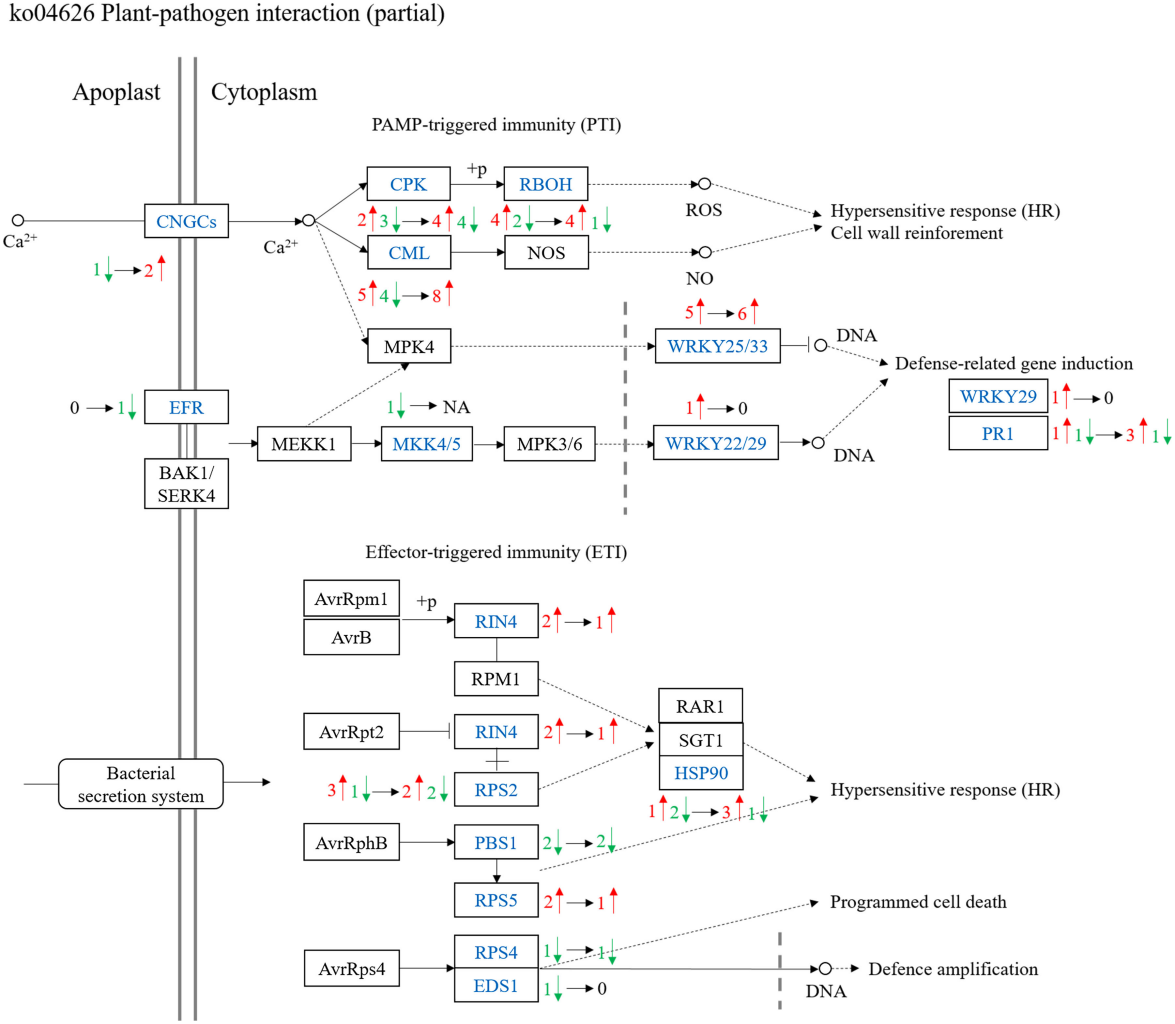
Comparative transcriptomic analysis of the plant-pathogen interaction pathway (ko04626) between HYZ5R (R) and HYZ62 (S). A simplified schematic diagram illustrating part of the plant-pathogen interaction pathway (ko04626) is presented, based on the KEGG pathway map (https://www.kegg.jp/pathway/ko04626). DEGs in HYZ5R (R) compared to HYZ62 (S) are labeled in blue text. The number of genes significantly up-regulated (red) and down-regulated (green) in HYZ5R (R) relative to HYZ62 (S) under the uninoculated condition is indicated on the left side of the arrows. The corresponding numbers of DEGs after inoculation with *P. brassicae* are shown on the right side of the arrows. HYZ62 and HYZ5R were two rapeseed varieties (**Supplementary Table S1**). Plants in the Uninoculated and Inoculated treatments were treated with 2 mL of water and resting spore suspension (1 × 10^8^ resting spores/mL), respectively.

Analysis of the plant-pathogen interaction pathway (ko04626) revealed distinct transcriptional patterns between HYZ5R (R) and HYZ62 (S) (**Figure 9**; **Dataset 3**). In uninoculated treatments, 46 DEGs were identified in HYZ5R (R) compared to HYZ62 (S) (27 up-regulated, 19 down-regulated). This number increased to 48 DEGs (35 up-regulated, 13 down-regulated) post-inoculation. A comparison of inoculated versus uninoculated plants showed that infection triggered the up-regulation of 11 and down-regulation of 12 genes in HYZ5R (R). In contrast, a more pronounced transcriptional shift occurred in HYZ62 (S), with 19 genes up-regulated and 36 down-regulated.

Analysis of PTI (pattern-triggered immunity) related genes (**Figure 9**; **Dataset 3**) showed that the number of genes with higher expression in HYZ5R (R) increased after inoculation, including *CNGC* (cyclic nucleotide-gated channel) (0 to 2), *CML* (calmodulin-like protein) (5 to 8), *WRKY25/33* (5 to 6), and *PR1* (pathogenesis-related protein 1) (1 to 3). Analysis of ETI (effector-triggered immunity) related genes showed that the number of *RIN4* (RPM1-interacting protein 4) and *RPS5* (resistance to *Pseudomonas syringae* 5) genes expressed significantly higher in HYZ5R (R) than in HYZ62 (S) decreased from two to one per gene family after inoculation. Meanwhile, the expression of *HSP90* (heat shock protein 90) (1 to 3) increased. In marked contrast, several defense-associated genes, such as *EFR* (EF-Tu receptor) and *MKK4/5* (mitogen-activated protein kinase kinase 4/5) (PTI-related), as well as *PBS1* (AvrPphB susceptible 1), *RPS4* (resistance to *P. syringae 4*), and *EDS1* (enhanced disease susceptibility 1) (ETI-related), exhibited elevated expression in HYZ62 (S).

#### 3.4.2 DEGs in the secondary metabolite biosynthesis pathways related to plant defense

We analyzed 199 DEGs involved in defense-related secondary metabolite biosynthesis pathways (**Dataset 4**). These included phenylpropanoid (ko00940, 147, 73.87%), glucosinolate (ko00966, 17, 8.54%), sesquiterpenoid and triterpenoid (ko00909, 10, 5.03%), diterpenoid (ko00904, 10, 5.03%), flavonoid (ko00941, 9, 4.52%), monoterpenoid (ko00902, 5, 2.51%), flavone and flavonol (ko00944, 1, 0.50%) biosynthesis pathways. Overall, inoculation led to significant down-regulation of genes in these defense-related pathways in both HYZ5R (R) and HYZ62 (S). This suppression was particularly evident in phenylpropanoid, flavonoid, and various terpenoid biosynthesis pathways.

The phenylpropanoid biosynthesis pathway (ko00940) was the most extensively and severely affected metabolic pathway in both HYZ5R (R) and HYZ62 (S) upon inoculation. In HYZ5R (R), nine genes were up-regulated, and 78 were down-regulated compared to its uninoculated control. In HYZ62 (S), six genes were up-regulated, and 74 were down-regulated. Notably, even under the uninoculated condition, 37 genes showed higher expression in HYZ5R (R) than in HYZ62 (S), while 13 were lower. After inoculation, 33 genes remained higher in HYZ5R (R), and 13 were lower. This suggests that HYZ5R (R) has a greater number of genes in this pathway that maintain higher expression levels than HYZ62 (S).

## 4 Discussion

### 4.1 The relationship between soil physicochemical properties and the occurrence of clubroot disease

The soil physicochemical properties not only provide favorable or unfavorable growth environments for soil-borne pathogens but also constitute the fundamental environmental conditions for maintaining soil microbial balance. Their deterioration may impair the disease resistance of plants.

Electrical conductivity (EC), a non-specific indicator of the total ion concentration in soil solution, is influenced by soil texture, water content, temperature, plant roots, and microbial activities (Corwin and Yemoto, 2020; Qadir et al., 2007; Uroz et al., 2009). Plant roots promote ionic dynamics in the rhizosphere through respiration, CO_2_ release, proton secretion, and the exudation of organic compounds (Qadir et al., 2007). Soil microorganisms, via mechanisms such as acidolysis, complexation, and redox reactions, can mobilize nutrient ions from minerals, thereby increasing ion concentrations in the soil solution (Uroz et al., 2009). In plant disease research, the relationship between EC and disease severity has been inconsistently reported. In studies on *Fusarium* wilt of banana, Domínguez et al. (2001) and Li et al. (2018) found that suppressive soils generally exhibit higher EC, which may be related to more active root function and ion transport. Similarly, this study found that the soil EC of HYZ5R (R) was significantly higher than that of HYZ62 (S). This difference may be associated with the less severe disease, and potentially more active root function in the resistant variety. However, Liao et al. (2022) and Arif et al. (2021) reported that the higher the severity of clubroot disease, the higher the soil EC or ion concentration in hydroponic solutions, explaining that this results from electrolyte leakage due to root tissue damage or impaired root uptake of water and salts, leading to relative ion accumulation. These contrasting findings indicate that the relationship between total soil EC and clubroot severity is not simply linear. Future research should clarify differences in ion composition under high EC conditions and consider how variations in soil microbial communities contribute to changes in specific ion concentrations, in order to elucidate the relationship between EC and clubroot occurrence more accurately.

The nutrient status of soil directly influences plant growth, yet the relationship between soil nutrients and the occurrence of soil-borne diseases remains unclear. The results of this study (**Figure 3**) indicated that both resistant and susceptible rapeseed exhibited a significant reduction in SOC (soil organic carbon) content after infection by *P. brassicae*. However, there was no significant difference between the two varieties. The Spearman correlation analysis (**Supplementary Figure S3**) further revealed that SOC was significantly negatively correlated with DI and DSI of clubroot disease. This finding aligns with some previously reported literature. Cao et al. (2022) also found a significant negative correlation between SOC and the severity of bacterial wilt disease, with SOC content being significantly higher in suppressive soils compared to conducive soils. Similarly, Adiobo et al. (2007) reported that high organic matter content (calculated as SOC) was a key factor in suppressing cocoyam root rot caused by *Pythium myriotylum* in andosols, and there was also a significant negative correlation between SOC and the severity of cocoyam root rot. High SOC content generally implies high soil microbial activity. In suppressive andosols, the abundances of total heterotrophic bacteria, fluorescent pseudomonads, and *Trichoderma* spp. were significantly higher than in conducive soils. The results of microbial community composition in this study were also partly consistent with these findings, showing that the abundance of the *Trichoderma* genus was significantly negatively correlated with DI and DSI of clubroot disease (**Supplementary Figure S3**). The relative abundance of the *Trichoderma* genus in HYZ5R (R) was significantly higher than in HYZ62 (S), although the difference between the two varieties became insignificant after infection by *P. brassicae*.

In addition, in this study, the AN (alkali-hydrolysable nitrogen) content of HYZ62 (S) significantly decreased after infection by *P. brassicae*, while the change in HYZ5R (R) was not significant. Spearman correlation analysis revealed a significant negative correlation between AN and the DI as well as the DSI of clubroot disease. This is consistent with the findings of Adiobo et al. (2007) that a higher soil nitrogen content was associated with reduced severity of cocoyam root rot. However, the general understanding is that high nitrogen supply typically tends to increase disease severity, but different nitrogen forms in soil may lead to varying results. Therefore, it remains unclear whether the significantly higher AN content in HYZ5R (R) compared to HYZ62 (S) after inoculation, coupled with the opposite disease severity, is related to different nitrogen forms. Finally, after infection by *P. brassicae*, the AK content in HYZ5R (R) was significantly higher than that in the uninoculated treatment, but there was no significant correlation between AK and DI/DSI of clubroot disease. Similarly, the study by Cao et al. (2022) also indicated that there was no significant correlation between AK and the severity of bacterial wilt disease.

### 4.2 The relationship between rhizosphere microbes and the occurrence of clubroot disease

Soil microbial diversity and composition are generally considered important factors in suppressing soil-borne diseases, influenced by various biotic and abiotic factors, including soil physicochemical properties, plant root exudates, and plant genotypes (Berendsen et al., 2012; Zhang et al., 2024).

The classical ecological perspective holds that a higher α-diversity of soil microorganisms corresponds to greater disease-suppressive capacity (Garbeva et al., 2004). This view has been confirmed in comparative studies of suppressive and conducive soils. For example, Kang et al. (2024) found that the fungal Shannon index and bacterial richness were significantly higher in clubroot-suppressive soils than in conducive soils. Li et al. (2024) also reported that in the tobacco root rot system caused by *Fusarium solani*, the fungal Shannon and Simpson indices were significantly higher in suppressive soils. However, no difference was observed in bacterial community α-diversity. When comparing plant genotypes with different resistance backgrounds within the same soil, the relationship between microbial α-diversity and disease occurrence becomes inconsistent. For instance, Xi et al. (2023) observed that after inoculation with *P. brassicae*, the susceptible pakchoi variety showed higher bacterial and fungal ASV numbers and α-diversity indices compared to the resistant variety. Meng et al. (2019) also found that the rhizobacterial richness and diversity were higher in *Fusarium oxysporum*-infected plants than in healthy plants. In this study, the rhizobacterial and fungal α-diversity indices were also significantly higher in HYZ62 (S) than in HYZ5R (R), consistent with these findings. Conversely, Deng et al. (2025) found that inoculation with *P. brassicae* did not cause significant changes in the rhizobacterial α-diversity of the same variety. In our study, no significant changes in bacterial or fungal α-diversity were observed between inoculated and uninoculated treatments of the two rapeseed varieties, except for a significant increase in the fungal sobs index in HYZ5R (R), which aligns with the results of Deng et al. (2025). In light of these results, we propose that, compared with α-diversity alone, microbial composition and function may have a greater influence on disease occurrence—a perspective also reflected in Garbeva et al. (2004).

Differences in microbial composition are primarily captured through β-diversity analysis, and the influence of resistant and susceptible varieties on rhizosphere microbial assembly has been well documented (Bakker et al., 2018; Kwak et al., 2018; Mendes et al., 2018). The β-diversity analysis (PCoA and NMDS) in this study revealed that the bacterial and fungal communities of HYZ5R (R) and HYZ62 (S) were significantly different (**Figure 5**), which aligns with Deng et al. (2025), who reported that variety type was the primary driver of rhizosphere bacterial β-diversity. We next discuss the detailed differences in bacterial and fungal composition at the genus level.

By comparing the rhizosphere bacterial and fungal genus compositions of resistant and susceptible rapeseed varieties, this study identifies microbial taxa whose relative abundances are associated with clubroot resistance. The analysis of bacterial genus differences revealed that, in the uninoculated condition, the relative abundances of *Sphingomonas*, *Humibacter*, and *Massilia* were significantly higher in HYZ5R (R) than in HYZ62 (S). After inoculation, the relative abundances of *Burkholderia-Caballeronia-Paraburkholderia*, *Humibacter*, and *Dyella* were significantly higher in HYZ5R (R) than in HYZ62 (S) (**Supplementary Table S5**), and these genera were significantly negatively correlated with the incidence and severity of clubroot disease (**Supplementary Figure S3**). Liu et al. (2024) and their cited references suggest that *Burkholderia-Caballeronia-Paraburkholderia* and *Dyella* have biocontrol potential against plant diseases and can rapidly respond to *Tobacco mosaic virus* (TMV) infection. However, in this study, the relative abundances of these genera significantly decreased after *P. brassicae* inoculation, contrary to the trends reported by Liu et al. (2024). Still, they remained higher in HYZ5R (R) than in HYZ62 (S). Additionally, the changes in *Dyella* abundance in this study are consistent with Xi et al. (2023), who reported a significant decrease in *Dyella* abundance after *P. brassicae* inoculation, and with Meng et al. (2019), who found lower *Dyella* abundance in the rhizosphere of diseased and dead watermelon plants compared to healthy ones. Therefore, the higher relative abundances of *Burkholderia-Caballeronia-Paraburkholderia* and *Dyella* in the rhizosphere of HYZ5R (R) suggest a possible association with clubroot resistance. Whether these bacteria contribute actively to disease suppression or their enrichment is a consequence of reduced disease severity remains to be determined. Functional validation through culture-based assays or synthetic community experiments is needed to establish causality.

Limited information is available on the roles of *Humibacter* and *Massilia* in plant disease resistance. Cordero-Elvia et al. (2024) found that the relative abundance of *Massilia* was higher in samples treated with *P. brassicae* strain B than in those treated with strain A, and its abundance was lower in susceptible canola at 21 days post-inoculation and in both canola varieties at 35 days post-inoculation. This study’s results are partially consistent, as *Massilia* abundance was higher in uninoculated rapeseed varieties and significantly decreased in both varieties 28 days post-inoculation.

*Bacillus*, as an important biocontrol bacterium, has been widely reported in the control of clubroot. Zhu et al. (2020) isolated two biocontrol strains, *B. velezensis* (F85) and *B. amyloliquefaciens* (T113), from the rhizosphere of *Brassica napus*, with both exhibiting a control efficacy of over 80% against clubroot. Arif et al. (2021) obtained *B. cereus* (MA-12) from the rhizosphere of *Brassica campestris* sp. *chinensis*, which showed a 73.4% control effect on clubroot. In addition, Cordero-Elvia et al. (2024) detected *Bacillus* in the rhizosphere of both resistant and susceptible canola. Although they did not explicitly analyze the differences in the relative abundance of *Bacillus* between the two cultivars, observations from their abundance stacked plots revealed that the relative abundance of *Bacillus* in the rhizosphere of the susceptible canola (12PH0244) decreased significantly at 35 days post-inoculation. Our finding, that the relative abundance of *Bacillus* in the rhizosphere of HYZ62 (S) decreased by 30.36% after infection, was consistent with their results. Therefore, we propose that the biocontrol efficacy of *Bacillus* may depend on its ability to colonize and proliferate in the rhizosphere. The reduction in *Bacillus* abundance in the rhizosphere of susceptible cultivars may be linked to disease-induced changes in root exudation, but this possibility requires experimental validation through root exudate profiling and controlled inoculation studies.

Regarding fungal genus differences, the most abundant fungal genera in the rhizosphere of both resistant and susceptible rapeseed varieties were *Trichoderma* (average relative abundance: 16.97%), *Penicillium* (9.04%), and *Fusarium* (5.22%). These findings are similar to those of Cordero-Elvia et al. (2024), who reported *Fusarium* (3%) as one of the most abundant fungal genera in samples treated with *P. brassicae* strains A and B, and *Trichoderma* (3%) as one of the most abundant genera in rhizosphere samples treated with strain B. These results suggest that *Trichoderma* and *Fusarium* are prominent members of the rhizosphere microbial communities in the context of clubroot disease, and their potential functions warrant further investigation. *Trichoderma*, a well-known biocontrol agent, has been shown to effectively suppress clubroot disease in multiple strains, such as *T. viride* (TR-7) (Arif et al., 2023), *T. Hz36* and *Hk37* (Zhao et al., 2022), and *T. harzianum* (Li et al., 2020). In this study, the relative abundance of *Trichoderma* in the rhizosphere of uninoculated HYZ5R (R) was significantly higher than in HYZ62 (S) (**Supplementary Table S5**), and this abundance was significantly negatively correlated with clubroot severity (**Supplementary Figure S3**). Although its relative abundance decreased after inoculation, it remained higher than that in HYZ62 (S). Given the reported biocontrol activity of *Trichoderma* against clubroot, this pattern raises the possibility that *Trichoderma* contributes to resistance in HYZ5R (R). However, direct evidence, such as isolation of clubroot-suppressive *Trichoderma* strains from this system or inoculation assays, is required to confirm a functional role. *Fusarium*, a ubiquitous soil-borne plant pathogenic fungus, significantly impacts plant health. However, the relationship between rhizosphere *Fusarium* abundance and clubroot disease occurrence has not been reported. This study found that *Fusarium* had the highest relative abundance in Ino_HYZ62 (S), but no significant differences were observed among all treatments. These results align with Cordero-Elvia et al. (2024), who reported minor differences in *Fusarium* abundance in the rhizosphere of resistant and susceptible canola but significant differences in root tissues. This suggests that *Fusarium* may be evenly distributed in the rhizosphere soil, but its colonization and activity in roots may be significantly influenced by host genotype. Further validation is needed to confirm the status of *Fusarium* in the roots of HYZ5R (R) and HYZ62 (S).

However, the association of these genera, along with others not mentioned, with clubroot disease development still needs further exploration. The composition of species within the same genus may more directly influence clubroot disease development, but this also needs further verification. Taxonomic inference at the genus level may obscure strain-level functional variation; closely related strains can have opposing effects on plant health. Future studies should combine amplicon sequencing with metagenomics or culture-dependent methods to identify active biocontrol strains and establish causality.

### 4.3 The relationship between DEGs in the transcriptome and the pathogenesis of clubroot

#### 4.3.1 DEGs in the plant-pathogen interaction pathway

The plant-pathogen interaction pathway reveals complex molecular cross-talk between plants and pathogens, which is critical for understanding disease development. Studying this pathway provides insights into plant immunity and pathogen virulence mechanisms (Jones and Dangl, 2006). In this study, the plant-pathogen interaction pathway reveals that HYZ5R (R) exhibits weaker transcriptional responses but maintains higher basal expression of *RBOHs*, *RIN4*, *WRKYs*, *PR1*, *RPS2*, and *RPS5*, which may be associated with its resistance. In contrast, key genes (*CNGCs*, *CMLs*, *RBOHs*, *PR1*, *RPS2*) were suppressed in HYZ62 (S). This down-regulation is consistent with its susceptibility phenotype. However, the observed suppression of these genes in HYZ62 (S) and their potential role in susceptibility requires further investigation.

##### 4.3.1.1 Calcium signaling in clubroot resistance

Calcium signaling plays a pivotal role in plant disease resistance (Wang et al., 2023). Upon pathogen invasion, calcium signaling is triggered, where CNGCs (Cyclic Nucleotide-Gated Channels) mediate the influx of extracellular Ca^2+^ into the cytosol, altering intracellular calcium concentrations. This process may also be modulated by CALM (Calmodulin) and CMLs (Calmodulin-like proteins), which directly bind Ca^2+^ and regulate downstream target proteins. CPKs (Calcium-Dependent Protein Kinases), as downstream effectors of calcium signaling, are activated by the CALM-Ca^2+^ complex and phosphorylate various targets, including defense-related genes (e.g., *PR* genes), thereby contributing to disease resistance. CPKs may also interact with other signaling pathways, such as the MAPK cascade (Adhikary et al., 2022; Luo et al., 2018).

In this study, we analyzed 47 calcium signaling-related DEGs. Among them, 21 genes were significantly down-regulated in the cultivar HYZ62 (S) upon *P. brassicae* infection, whereas only three genes were down-regulated in the cultivar HYZ5R (R). These findings are consistent with Mei et al. (2019), who reported similar trends during early infection stages (0–6 dpi) in *B. napus*. However, unlike Mei et al. (2019), our study focused on late-stage infection (28 dpi). Thus, while we cannot confirm whether HYZ5R (R) rapidly activates calcium signaling at early stages (as observed in ZHE-226), we demonstrate that calcium signaling remains suppressed in HYZ62 (S) even at late infection stages.

Specifically, HYZ62 (S) exhibited significant down-regulation of 8 *CNGCs* and 12 *CMLs*, which may explain its impaired calcium signaling. These results align with Adhikary et al. (2022), who observed reduced protein levels of CMLs, CALM, and CPKs in susceptible canola cultivars at 7, 14, and 21 dpi. However, our study only examined transcriptional changes at 28 dpi. Collectively, these findings suggest that calcium signaling suppression begins early in susceptible cultivars and persists until late infection stages, which may be linked to their susceptibility. Intriguingly, 7 *CPK* genes were significantly up-regulated in HYZ62 (S) (versus only 3 in HYZ5R (R)). The regulatory mechanisms underlying this observation and whether CPK protein levels correlate with transcript abundance require further investigation.

Zhou et al. (2020) highlighted that Ca^2+^ activates *RBOHs* (Respiratory Burst Oxidase Homologs), promoting ROS production. Adhikary et al. (2022) also reported higher peroxidase activity in resistant cultivars, suggesting interplay between calcium signaling and ROS in clubroot defense. In our study, 14 differentially expressed *RBOHs* were identified. Both HYZ62 (S) and HYZ5R (R) showed up-regulation of 3 *RBOHs* upon infection, but HYZ62 (S) additionally down-regulated 4 *RBOHs* (versus only 2 in HYZ5R (R)). Moreover, HYZ5R (R) exhibited higher basal expression of *RBOHs* than HYZ62 in both inoculated and uninoculated conditions. We hypothesize that the higher expression of *RBOHs* in HYZ5R (R) may be associated with stronger ROS production and enhanced clubroot resistance, though experimental validation is needed.

##### 4.3.1.2 PTI/ETI and defense-related gene expression

Plants defend against pathogens through a two-layered immune system (Jones and Dangl, 2006). The first layer, PTI (PAMP-Triggered Immunity), is activated when PRRs (Pattern Recognition Receptors) recognize conserved microbial patterns. Successful pathogens deliver effector proteins into plant cells to suppress PTI, leading to ETS (effector-triggered susceptibility). In response, plants have evolved resistance (R) proteins, mostly of the NB-LRR proteins, that recognize these effectors either directly or indirectly, activating the second layer, ETI (Effector-Triggered Immunity), which often culminates in a HR (Hypersensitive Response) at the infection site. Upon PAMP recognition, signals are transduced through MAPK cascades, activating transcription factors such as *WRKYs*, and defense genes such as *PR1* (Zhou et al., 2020; Mei et al., 2019), and *FRK1* (PTI marker gene) (Hsu et al., 2013). Calcium signaling also plays a role in PTI signal transduction, which we have discussed before. In the “guard model” of ETI signaling, NB-LRR proteins monitor the integrity of host proteins targeted by effectors, activating ETI. For example, in the *Arabidopsis*-*P. syringae* interaction system, it has been found that the effector AvrPphB cleaves the PTI signaling component PBS1 (AvrPphB Susceptible 1) to suppress PTI, while in hosts containing the RPS5 resistance protein, this cleavage process activates RPS5-mediated ETI (Hou et al., 2011). Additionally, effectors AvrRpt2 and AvrRpm1/AvrB cleave and phosphorylate RIN4 (a negative regulator of PTI), respectively. This process is recognized and activates ETI in hosts containing RPS2 and RPM1 resistance proteins, respectively (Hou et al., 2011; Mackey et al., 2002; Kim et al., 2005).

In clubroot research, the activation of host PTI and ETI is also considered a major mechanism for defending against *P. brassicae* infection. Luo et al. (2018) demonstrated that treatment with the biocontrol agent *Zhihengliuella aestuarii* up-regulated the expression of *PRRs* (such as *BAK1*, *CERK1*, and *CEBiP*) as well as resistance proteins RPS4 and RPS5 in *B. juncea* var. *tumida* Tsen. Shah et al. (2020), in a transcriptomic comparison between *B. napus* lines carrying *PbBa8.1* or *CRb* resistance genes and susceptible varieties, found that in resistant varieties, PTI-related genes, including key SA pathway genes (e.g., *NPR1*, *TGA*, *PR1*), ROS-generating genes (e.g., *RBOH*), antioxidant enzyme genes (e.g., *GST*, *SOD*), and transcription factors (e.g., *WRKY*, *ERF*) were significantly enhanced. Additionally, the expression of ETI-related resistance proteins RPS2 and RPS3 was significantly up-regulated. Studies by Mei et al. (2019) and Zhou et al. (2020) on resistant and susceptible rapeseed varieties also support this view, with differences observed in the specific *PRRs*, transcription factors, and resistance genes involved. Moreover, plants employ some non-classical PTI and ETI defense responses against clubroot. Wu et al. (2025b) identified the susceptibility gene *GSL5* in plants; the clubroot effector *PbPDIa* targets this gene to inhibit the JA defense pathway, promoting susceptibility. The *gsl5* mutant exhibited disease resistance responses at 12 days post-inoculation with clubroot, including up-regulation of *PR1*, *PR2*, and *PR5* genes, accumulation of lignin and callose, and increased cell death. Wang et al. (2023) discovered the broad-spectrum clubroot resistance gene *WTS* (*WeiTsing*) in the *Arabidopsis* resistant material Est-1. This gene blocks further invasion of the pathogen into the stele by inducing pericycle-specific calcium signaling and activating defense genes such as *PR2* and *PR5*.

In this study, both HYZ5R (R) and HYZ62 (S) exhibited down-regulation of *FLS2* upon infection. FLS2 is a well-characterized PRR, and its suppression is a common effector strategy to inhibit PTI (Hou et al., 2011). This suggests that *P. brassicae* effectors may suppress PTI in both cultivars. Downstream of PTI signaling, HYZ62 (S) showed up-regulation of *MEKK1*, *MEK1*, two *WRKY22* genes, and finally one *FRK1* gene up-regulated and one *PR1* gene down-regulated. This activation in HYZ62 (S) may represent an attempt to mount a PTI response, but it was ultimately insufficient to prevent susceptibility. In contrast, HYZ5R (R) exhibited minimal changes in these PTI-related genes upon infection. However, its basal expression levels of *WRKYs* and *PR1* were consistently higher than those in HYZ62 (S), which may be associated with its resistance. In ETI-related genes, HYZ62 (S) showed significant down-regulation of three *RPS2* genes upon infection. In HYZ5R (R), two *RPS2* genes were significantly down-regulated, while one *RPS2* gene was significantly up-regulated. Comparison between the two varieties revealed that, regardless of inoculation status, the expression levels of *RPS2*, *RPS5*, and *RIN4* were higher in HYZ5R (R) than in HYZ62 (S). This aligns with partial findings from Shah et al. (2020); Mei et al. (2019), and Zhou et al. (2020). Overall, the relatively small transcriptional changes in PTI- and ETI- related genes upon infection in HYZ5R (R) may be related to our observation time point (28 dpi) being at a late infection stage rather than early stages. Then, their consistently higher expression levels in HYZ5R (R) both under inoculation and uninoculation conditions may be associated with its resistance.

Nevertheless, the role of *FRK1* (enhances resistance to *P. syringae* in flooded Arabidopsis) (Hsu et al., 2013) in HYZ62 (S) warrants further investigation, and manipulation of this gene could potentially alter host resistance. Additionally, Wei et al. (2021) identified *RIN4* as one of the 15 hub genes that were highly correlated with clubroot resistance in Chinese cabbage at multiple time points (3, 9, and 20 days post-inoculation) within a co-expression network. Therefore, how resistant materials utilize the hub role of *RIN4* to regulate gene expression and enhance disease resistance could be a future research direction.

##### 4.3.1.3 Role of *EDS1* in clubroot resistance

*EDS1* is a key disease resistance gene. Atem et al. (2024) functionally validated its role in *B. napus*, showing that overexpression enhances clubroot resistance, whereas RNAi increases susceptibility. In our study, two *EDS1* genes were down-regulated in HYZ62 (S) but unchanged in HYZ5R (R), which raises the possibility that *EDS1* may be associated with susceptibility in HYZ62 (S), although its role in HYZ5R (R) resistance remains unclear. As an upstream regulator of SA signaling, *EDS1* influences downstream defense gene *PR1*, whose expression trends were similar to those of *EDS1*.

#### 4.3.2 DEGs in the biosynthesis pathways of defense-related secondary metabolites

Within the complex network of plant-pathogen interactions, although secondary metabolites are synthesized in small quantities, they play a crucial role in protecting plants against various biotic and abiotic stresses (Goura et al., 2025). Among them, the phenylpropanoid biosynthesis pathway, which is involved in the synthesis of essential metabolites such as lignin and flavonoids, serves as a central hub for plant resistance against *P. brassicae* infection (Zhang et al., 2023; Irani et al., 2018, 2019). These studies investigated infection stages ranging from early to late phases. However, the findings of Ciaghi et al. (2019) revealed that, compared to healthy roots from the same plant, *P. brassicae* infection led to a significantly lower expression level of genes in this pathway in galls. The results of our study also found that infection by *P. brassicae* broadly caused significant down-regulation of genes in this pathway (compared to healthy plants) in both the HYZ5R (R) and HYZ62 (S), which is relatively consistent with the results of Ciaghi et al. (2019), but opposite to those of Irani et al. (2019).

Nevertheless, in this study, the expression levels of more genes in this pathway were significantly higher in both inoculated and uninoculated HYZ5R (R) plants than in HYZ62 (S). This observation is consistent with the proposed role of these pathway genes in clubroot resistance. The observed suppression of the phenylpropanoid metabolic pathway following infection may reflect a pathogen’s immune evasion strategy, potentially limiting the synthesis of defensive compounds such as lignin and flavonoids. However, this hypothesis requires functional validation.

Furthermore, the expression of multiple genes in the glucosinolate biosynthesis pathway was also significantly down-regulated at 4 weeks post-inoculation with *P. brassicae* in both HYZ5R (R) and HYZ62 (S). These results are relatively consistent with some findings from Irani et al. (2018) and Blicharz et al. (2025). The suppression of secondary metabolite biosynthetic pathways by *P. brassicae* infection has been observed in both resistant and susceptible varieties. Whether this suppression represents a pathogen-driven process that facilitates infection, or a plant-mediated response that reallocates resources under stress, remains to be determined. Functional studies are needed to distinguish between these possibilities.

## 5 Conclusion

Based on the analysis of soil physicochemical properties, rhizosphere bacterial and fungal communities, and plant transcriptomes of HYZ5R (R) and HYZ62 (S) in response to clubroot, we found that HYZ5R (R) exhibits higher electrical conductivity and a more stable alkali-hydrolysable nitrogen level. Although its rhizosphere bacterial and fungal α-diversity indices were significantly lower than those of HYZ62 (S), the composition of its rhizosphere microbial community revealed that several bacterial and fungal genera previously reported as potential biocontrol agents had higher relative abundances in HYZ5R (R). Although HYZ62 (S) had a higher relative abundance of *Bacillus* under the uninoculated condition, its relative abundance significantly decreased after inoculation. We speculate that different varieties are associated with distinct groups of specific microorganisms, and the stability of these microbial communities may be associated with their disease resistance. In response to *P. brassicae* infection, HYZ5R (R) exhibited significantly higher expression levels of most genes involved in pathogen-host interaction pathways and defense-related secondary metabolite biosynthesis compared to HYZ62 (S). However, pathogen infection also strongly suppressed the expression of genes in these defense-related secondary metabolite pathways in both varieties. This suggests that HYZ5R (R) may employ a balancing mechanism to coordinate its resistance, in which the function of *RIN4* warrants further investigation. In HYZ62 (S), these down-regulated genes could be potential targets for resistance improvement through genetic modification. Genes up-regulated in HYZ62 (S), such as *FRK1* (an early PTI marker gene), should also be examined for their potential roles in either susceptibility or resistance. Although HYZ62 (S) exhibits susceptibility under high concentrations of *P. brassicae* infection, it does not reach a highly susceptible state. This may be associated with its high α-diversity, the relatively high abundance of *Bacillus*, and the up-regulation of *WRKY* transcription factors and the *FRK1* gene, though further verification is required.

## Data Availability

The raw sequencing data have been deposited in the NCBI Sequence Read Archive (SRA) under BioProject accession numbers PRJNA1336419 (for rhizosphere soil bacteria and fungi) and PRJNA1342181 (for the rapeseed root transcriptome).

## Supplementary material

Supplementary Figure S1 KEGG pathway enrichment analysis of DEGs from four comparative groups. The four comparisons are: Ino_HYZ62 vs Uni_HYZ62, Ino_HYZ5R vs Uni_HYZ5R, Uni_HYZ5R vs Uni_HYZ62, Ino_HYZ5R vs Ino_HYZ62. The bubble plot displays the top significantly enriched KEGG pathways for each comparison. The size of the bubble represents the number of DEGs enriched in the pathway, and the color indicates the enrichment factor. Pathways marked with a red triangle were selected for detailed subsequent analysis. HYZ62 and HYZ5R were two rapeseed varieties (**Supplementary Table S1**). Plants in the Uninoculated (Uni) and Inoculated (Ino) treatments were treated with 2 mL of water and resting spore suspension (1 × 10^8^ resting spores/mL), respectively.

Supplementary Figure S2 Validation of RNA-seq data by qPCR for six selected genes. Bar and line graph showing the log_2_FC change of gene expression derived from the RNA-seq and qPCR, respectively. Gene expression levels in qPCR were normalized to *BnTUB* (*B. napus* tubulin beta chain, *LOC111213844*). Detailed gene information is available in **Supplementary Table S2**. The experimental treatments included: inoculated HYZ62 (I62), uninoculated HYZ62 (U62), inoculated HYZ5R (I5R), and uninoculated HYZ5R (U5R). HYZ62 and HYZ5R were two rapeseed varieties (**Supplementary Table S1**). Plants in the Uninoculated (Uni) and Inoculated (Ino) treatments were treated with 2 mL of water and resting spore suspension (1 × 10^8^ resting spores/mL), respectively.

Supplementary Figure S3 Spearman correlation heatmap between rhizosphere microbial taxa, clubroot severity, and soil physicochemical properties. **(A)** Phylum-level and **(B)** genus-level bacterial and fungal taxa. The matrices display Spearman’s rank correlation coefficients (r) between microbial taxa and disease parameters (DI, disease incidence; DSI, disease index) or soil physicochemical properties (pH; EC, electrical conductivity; SOC, soil organic carbon; AN, alkali-hydrolysable nitrogen; AP, available phosphorus; AK, available potassium. Only taxa with a relative abundance > 1% in at least one treatment are shown. Asterisks indicate significant differences (**P* < 0.05, ***P* < 0.01, ****P* < 0.001). The dendrogram on the left represents hierarchical clustering of the disease and soil parameters based on their correlation patterns with the microbiota. The color scale indicates the correlation coefficient, with purple and orange representing negative and positive correlations, respectively.Table legends.

Supplementary Table S1 Names and sources of the six rapeseed varieties tested.

Supplementary Table S2 The information on qPCR primers.

Supplementary Table S3 Incidence and disease index of clubroot on six rapeseed varieties. HYZ62, HS5R, YW, FY135R, HYZ160R, HYZ5R were six rapeseed varieties (**Supplementary Table S1**). Values represent the mean of three biological replicates ± SE. Plants were treated with 2 mL resting spore suspension (1 × 10^8^ resting spores/mL). All data were collected at four weeks post-inoculation. Different lowercase letters indicate significant differences based on one-way ANOVA (*P* < 0.05, LSD test).

Supplementary Table S4 Relative abundances of bacteria and fungi at the phylum level. Values represent the mean of six biological replicates; error bars represent SE. Asterisks indicate significant differences between Ino_HYZ5R and Ino_HYZ62, and between Uni_HYZ5R and Uni_HYZ62 (Student’s t-test **P* < 0.05, ***P* < 0.01, ****P* < 0.001). Bold text indicates a significant difference (Student’s t-test, *P* < 0.05) for the pairwise comparisons between Ino_HYZ5R and Uni_HYZ5R, and between Ino_HYZ62 and Uni_HYZ62. The experimental treatments included: inoculated HYZ62 (Ino_HYZ62), uninoculated HYZ62 (Uni_HYZ62), inoculated HYZ5R (Ino_HYZ5R), and uninoculated HYZ5R (Uni_HYZ5R).

Supplementary Table S5 Relative abundances of bacteria and fungi at the genus level. Values represent the mean of six biological replicates; error bars represent SE. The experimental treatments included: inoculated HYZ62 (Ino_HYZ62), uninoculated HYZ62 (Uni_HYZ62), inoculated HYZ5R (Ino_HYZ5R), and uninoculated HYZ5R (Uni_HYZ5R). The significant differences between the two treatments were analyzed using the Student’s t-test.

DATASET 1 Summary of transcriptome sequencing and mapping statistics.

DATASET 2 Summary of high-throughput sequencing dataset.

DATASET 3 Differentially expressed genes were significantly enriched in the plant-pathogen interaction pathway (ko04626). The experimental treatments included: inoculated HYZ62 (Ino_HYZ62), uninoculated HYZ62 (Uni_HYZ62), inoculated HYZ5R (Ino_HYZ5R), and uninoculated HYZ5R (Uni_HYZ5R). These genes were screened based on KEGG enrichment analysis results. The log_2_FC values in red, green, and black indicate significant upregulation, downregulation, and insignificant changes in gene expression, respectively. “NA” stands for “not available”, indicating that no information was provided. Differential gene expression analysis was conducted using edgeR (version 3.42.4). Raw read counts were normalized using the TMM (trimmed mean of M-values) method to correct for library size differences. A negative binomial generalized linear model was fitted to the count data to estimate dispersion parameters and account for biological variability. log_2_FC was calculated from the model coefficients representing the contrast between treatment and control groups. Statistical significance was determined using edgeR’s exact test (for simple comparisons) or quasi-likelihood F-test (for complex designs), which are specifically designed for overdispersed count data. The resulting *P*-values were adjusted for multiple testing using the Benjamini-Hochberg procedure to obtain FDR.

DATASET 4 Differentially expressed genes in the biosynthesis pathways of defense-related secondary metabolites.The experimental treatments included: inoculated HYZ62 (Ino_HYZ62), uninoculated HYZ62 (Uni_HYZ62), inoculated HYZ5R (Ino_HYZ5R), and uninoculated HYZ5R (Uni_HYZ5R). These genes were screened based on KEGG enrichment analysis results. The log_2_FC values in red, green, and black indicate significant upregulation, downregulation, and insignificant changes in gene expression, respectively. “NA” stands for “not available”, indicating that no information was provided. Differential gene expression analysis was conducted using edgeR (version 3.42.4). Raw read counts were normalized using the TMM (trimmed mean of M-values) method to correct for library size differences. A negative binomial generalized linear model was fitted to the count data to estimate dispersion parameters and account for biological variability. log_2_FC was calculated from the model coefficients representing the contrast between treatment and control groups. Statistical significance was determined using edgeR’s exact test (for simple comparisons) or quasi-likelihood F-test (for complex designs), which are specifically designed for overdispersed count data. The resulting *P*-values were adjusted for multiple testing using the Benjamini-Hochberg procedure to obtain FDR.

## References

[B1] Adhikary D. Mehta D. Uhrig R. G. Rahman H. Kav N. N. V. (2022). A proteome-level investigation into Plasmodiophora brassicae resistance in Brassica napus canola. Front. Plant Sci. 13. doi: 10.3389/fpls.2022.860393. PMID: 35401597 PMC8988049

[B2] Adiobo A. Oumar O. Perneel M. Zok S. Höfte M. (2007). Variation of Pythium-induced cocoyam root rot severity in response to soil type. Soil Biol. Biochem. 39, 2915–2925. doi: 10.1016/j.soilbio.2007.06.012. PMID: 41940325

[B3] Agarwal A. Kaul V. Faggian R. Rookes J. E. Ludwig-Müller J. Cahill D. M. (2011). Analysis of global host gene expression during the primary phase of the Arabidopsis thaliana-Plasmodiophora brassicae interaction. Funct. Plant Biol. 38, 462–478. doi: 10.1071/fp11026. PMID: 32480901

[B4] Arif S. Liaquat F. Yang S. L. Shah I. H. Zhao L. N. Xiong X. . (2021). Exogenous inoculation of endophytic bacterium Bacillus cereus suppresses clubroot (Plasmodiophora brassicae) occurrence in Pak Choi (Brassica campestris sp. chinensis L.). Planta 253, 25. doi: 10.1007/s00425-020-03546-4. PMID: 33404767

[B5] Arif S. Munis M. F. H. Liaquat F. Gulzar S. Haroon U. Zhao L. N. . (2023). Trichoderma viride establishes biodefense against clubroot (Plasmodiophora brassicae) and fosters plant growth via colonizing root hairs in Pak Choi (Brassica campestris spp. chinesnsis). Biol. Control 183, 105265. doi: 10.1016/j.biocontrol.2023.105265, PMID: 38826717

[B6] Atem J. E. C. Gan L. C. Yu W. L. Huang F. Wang Y. Y. Baloch A. . (2024). Bioinformatics and functional analysis of EDS1 genes in Brassica napus in response to Plasmodiophora brassicae infection. Plant Sci. 347, 112175. doi: 10.1016/j.plantsci.2024.112175. PMID: 38986913

[B7] Bakker P. A. H. M. Pieterse C. M. J. de Jonge R. Berendsen R. L. (2018). The soil-borne legacy. Cell. 172, 1178–1180. doi: 10.1016/j.cell.2018.02.024. PMID: 29522740

[B8] Berendsen R. L. Pieterse C. M. J. Bakker P. A. H. M. (2012). The rhizosphere microbiome and plant health. Trends Plant Sci. 17, 478–486. doi: 10.1016/j.tplants.2012.04.001. PMID: 22564542

[B9] Bhering A. S. do Carmo M. G. F. Coelho I. S. Lima E. S. A. de Carvalho C. F. Saraiva A. L. R. F. . (2020). Soil management in a mountain agroecosystem and clubroot disease. Plant Pathol. 69, 302–309. doi: 10.1111/ppa.13123. PMID: 41940437

[B10] Blicharz S. Stefanowicz K. Truman W. Basińska-Barczak A. Singh D. Kasprzewska A. . (2025). Laser dissection‐assisted phloem transcriptomics highlights the metabolic and physiological changes accompanying clubroot disease progression in oilseed rape. Plant J. 121, e17156. doi: 10.1111/tpj.17156. PMID: 39575835 PMC11703547

[B11] Cao Y. F. Thomashow L. S. Luo Y. Hu H. W. Deng X. H. Liu H. J. . (2022). Resistance to bacterial wilt caused by Ralstonia solanacearum depends on the nutrient condition in soil and applied fertilizers: A meta-analysis. Agric. Ecosyst. Environ. 329, 107874. doi: 10.1016/j.agee.2022.107874. PMID: 41940325

[B12] Chai A. L. Xie X. W. Shi Y. X. Li B. J. (2014). Research status of clubroot (Plasmodiophora brassicae) on cruciferous crops in China. Can. J. Plant Pathol. 36, 142–153. doi: 10.1080/07060661.2013.868829. PMID: 41909888

[B13] Ciaghi S. Schwelm A. Neuhauser S. (2019). Transcriptomic response in symptomless roots of clubroot infected kohlrabi (Brassica oleracea var. gongylodes) mirrors resistant plants. BMC Plant Biol. 19, 288. doi: 10.1101/391516. PMID: 31262271 PMC6604361

[B14] Cordero-Elvia J. Galindo-González L. Fredua-Agyeman R. Hwang S. F. Strelkov S. E. (2024). Clubroot-induced changes in the root and rhizosphere microbiome of susceptible and resistant canola. Plants 13, 1880. doi: 10.3390/plants13131880. PMID: 38999720 PMC11244039

[B15] Corwin D. L. Yemoto K. (2020). Salinity: Electrical conductivity and total dissolved solids. Soil Sci. Soc Am. J. 84, 1442–1461. doi: 10.1002/saj2.20154. PMID: 41939252

[B16] Daval S. Gazengel K. Belcour A. Linglin J. Guillerm-Erckelboudt A. Y. Sarniguet A. . (2020). Soil microbiota influences clubroot disease by modulating Plasmodiophora brassicae and Brassica napus transcriptomes. Microb. Biotechnol. 13, 1648–1672. doi: 10.1111/1751-7915.13634. PMID: 32686326 PMC7415369

[B17] Deng Y. Wu W. X. Huang X. Q. Yang X. X. Yu Y. Y. Zhang Z. M. . (2025). Characterization of rhizosphere bacterial communities in oilseed rape cultivars with different susceptibility to Plasmodiophora brassicae infection. Front. Plant Sci. 15. doi: 10.3389/fpls.2024.1496770. PMID: 39834703 PMC11743679

[B18] Dixon G. R. (2009). The occurrence and economic impact of Plasmodiophora brassicae and clubroot disease. J. Plant Growth Regul. 28, 194–202. doi: 10.1007/s00344-009-9090-y. PMID: 41940407

[B19] Domínguez J. Negrín M. A. Rodríguez C. M. (2001). Aggregate water-stability, particle-size and soil solution properties in conducive and suppressive soils to fusarium wilt of banana from Canary Islands (Spain). Soil Biol. Biochem. 33, 449–455. doi: 10.1016/S0038-0717(00)00184-X

[B20] Garbeva P. van Veen J. A. van Elsas J. D. (2004). Microbial diversity in soil: Selection of microbial populations by plant and soil type and implications for disease suppressiveness. Annu. Rev. Phytopathol. 42, 243–270. doi: 10.1146/annurev.phyto.42.012604.135455. PMID: 15283667

[B21] Goura K. Legrifi I. Kallali N. S. Taoussi M. Kenfaoui J. Meddich A. . (2025). Beyond survival: The role of secondary metabolites in plant defense mechanisms. J. Crop Health 77, 121. doi: 10.1007/s10343-025-01183-z. PMID: 41940407

[B22] Hou S. Yang Y. Wu D. Zhang C. (2011). Plant immunity. Plant Signal. Behav. 6, 794–799. doi: 10.4161/psb.6.6.15143. PMID: 21494098 PMC3218475

[B23] Hsu F. C. Chou M. Y. Chou S. J. Li Y. R. Peng H. P. Shih M. C. (2013). Submergence confers immunity mediated by the WRKY22 transcription factor in Arabidopsis. Plant Cell 25, 2699–2713. doi: 10.1105/tpc.113.114447. PMID: 23897923 PMC3753392

[B24] Irani S. Todd C. D. Wei Y. Bonham-Smith P. C. (2019). Changes in phenylpropanoid pathway gene expression in roots and leaves of susceptible and resistant Brassica napus lines in response to Plasmodiophora brassicae inoculation. Physiol. Mol. Plant Pathol. 106, 196–203. doi: 10.1016/j.pmpp.2019.02.007. PMID: 41940325

[B25] Irani S. Trost B. Waldner M. Nayidu N. Tu J. Kusalik A. J. . (2018). Transcriptome analysis of response to Plasmodiophora brassicae infection in the Arabidopsis shoot and root. BMC Genomics 19, 23. doi: 10.1186/s12864-017-4426-7. PMID: 29304736 PMC5756429

[B26] Jones J. D. G. Dangl J. L. (2006). The plant immune system. Nature 444, 323–329. doi: 10.1038/nature05286. PMID: 17108957

[B27] Kang H. J. Lin Z. H. Yuan X. W. Shi Y. X. Xie X. W. Li L. . (2024). The occurrence of clubroot in cruciferous crops correlates with the chemical and microbial characteristics of soils. Front. Microbiol. 14. doi: 10.3389/fmicb.2023.1293360. PMID: 38260873 PMC10800485

[B28] Kim M. G. da Cunha L. McFall A. J. Belkhadir Y. DebRoy S. Dangl J. L. . (2005). Two Pseudomonas syringae type III effectors inhibit RIN4-regulated basal defense in Arabidopsis. Cell. 121, 749–758. doi: 10.3348/jkrs.1976.12.2.171. PMID: 15935761

[B29] Kwak M. J. Kong H. G. Choi K. Kwon S. K. Song J. Y. Lee J. . (2018). Rhizosphere microbiome structure alters to enable wilt resistance in tomato. Nat. Biotechnol. 36, 1100–1109. doi: 10.1038/nbt.4232. PMID: 30295674

[B30] Li Z. Deng Z. Chen S. Yang H. Zheng Y. Dai L. . (2018). Contrasting physical and biochemical properties of orchard soils suppressive and conducive to fusarium wilt of banana. Soil Use Manag. 34, 154–162. doi: 10.1111/sum.12390. PMID: 41940437

[B31] Li L. X. Luo Y. J. Chen B. Y. Xu K. Zhang F. G. Li H. . (2016). A genome-wide association study reveals new loci for resistance to clubroot disease in Brassica napus. Front. Plant Sci. 7. doi: 10.3389/fpls.2016.01483. PMID: 27746804 PMC5044777

[B32] Li Q. Nadil S. Zhou Y. W. Hou Z. K. Gong J. F. Liu J. . (2021). Breeding of a novel clubroot disease-resistant Brassica napus variety Huayouza 62R. Acta Agron. Sin. 47, 210–223. doi: 10.3724/sp.j.1006.2021.04086. PMID: 41207781

[B33] Li J. Philp J. Li J. Wei Y. Li H. Yang K. . (2020). Trichoderma harzianum inoculation reduces the incidence of clubroot disease in Chinese cabbage by regulating the rhizosphere microbial community. Microorganisms 8, 1325. doi: 10.3390/microorganisms8091325, PMID: 32878079 PMC7563613

[B34] Li B. L. Yang P. Feng Y. L. Du C. Y. Qi G. F. Zhao X. Y. (2024). Rhizospheric microbiota of suppressive soil protect plants against Fusarium solani infection. Pest Manage. Sci. 80, 4186–4198. doi: 10.1002/ps.8122. PMID: 38578633

[B35] Liao J. J. Luo L. Y. Zhang L. Wang L. Z. Shi X. D. Yang H. . (2022). Comparison of the effects of three fungicides on clubroot disease of tumorous stem mustard and soil bacterial community. J. Soils Sediments 22, 256–271. doi: 10.1007/s11368-021-03073-z. PMID: 41940407

[B36] Liu C. Feng Z. C. Xiao T. H. Ma X. M. Zhou G. S. Huang F. H. . (2019). Development, potential and adaptation of Chinese rapeseed industry. Chin. J. Oil Crop Sci. 41, 485–489.

[B37] Liu S. N. Xiao Z. P. Xiao Y. S. Liu T. B. Wu S. L. Ren Z. H. . (2024). Impact of soil sterilization on antagonistic efficiency against tobacco mosaic virus and the rhizosphere bacterial community in Nicotiana benthamiana. Rhizosphere 31, 100941. doi: 10.1016/j.rhisph.2024.100941. PMID: 41940325

[B38] Livak K. J. Schmittgen T. D. (2001). Analysis of relative gene expression data using real-time quantitative PCR and the 2^-ΔΔCT^ method. Methods 25, 402–408. doi: 10.1006/meth.2001.1262. PMID: 11846609

[B39] Luo Y. Dong D. Su Y. Wang X. Peng Y. Peng J. . (2018). Transcriptome analysis of Brassica juncea var. tumida Tsen responses to Plasmodiophora brassicae primed by the biocontrol strain Zhihengliuella aestuarii. Funct. Integr. Genomics 18, 301–314. doi: 10.1007/978-3-030-24716-4_7. PMID: 29564648

[B40] Mackey D. Holt B. F. H. III Wiig A. Dangl J. L. (2002). RIN4 interacts with Pseudomonas syringae type III effector molecules and is required for RPM1-mediated resistance in Arabidopsis. Cell 108, 743–754. doi: 10.1016/s0092-8674(02)00661-x. PMID: 11955429

[B41] Manzanares-Dauleux M. J. Delourme R. Baron F. Thomas G. (2000). Mapping of one major gene and of QTLs involved in resistance to clubroot in Brassica napus. Theor. Appl. Genet. 101, 885–891. doi: 10.1007/s001220051557. PMID: 41940407

[B42] Mei J. Guo Z. Wang J. Feng Y. Ma G. Zhang C. . (2019). Understanding the resistance mechanism in Brassica napus to clubroot caused by Plasmodiophora brassicae. Phytopathology 109, 810–818. doi: 10.1094/phyto-06-18-0213-r. PMID: 30614377

[B43] Mendes L. W. Raaijmakers J. M. de Hollander M. Mendes R. Tsai S. M. (2018). Influence of resistance breeding in common bean on rhizosphere microbiome composition and function. ISME J. 12, 212–224. doi: 10.1038/ismej.2017.158. PMID: 29028000 PMC5739014

[B44] Meng T. Z. Wang Q. J. Abbasi P. Ma Y. (2019). Deciphering differences in the chemical and microbial characteristics of healthy and Fusarium wilt-infected watermelon rhizosphere soils. Appl. Microbiol. Biotechnol. 103, 1497–1509. doi: 10.1007/s00253-018-9564-6. PMID: 30560450

[B45] Murakami H. Tsushima S. Shishido Y. (2000). Soil suppressiveness to clubroot disease of Chinese cabbage caused by Plasmodiophora brassicae. Soil Biol. Biochem. 32, 1637–1642. doi: 10.1016/s0038-0717(00)00079-1. PMID: 41334505

[B46] Neik T. X. Barbetti M. J. Batley J. (2017). Current status and challenges in identifying disease resistance genes in Brassica napus. Front. Plant Sci. 8. doi: 10.3389/fpls.2017.01788. PMID: 29163558 PMC5681527

[B47] Qadir M. Oster J. D. Schubert S. Noble A. D. (2007). “ Phytoremediation of sodic and saline-sodic soils,” in Elsevier. ( Academic Press), 197–247.

[B48] Ren Y. Yu G. Shi C. Liu L. Guo Q. Han C. . (2022). Majorbio Cloud: A one-stop, comprehensive bioinformatic platform for multiomics analyses. iMeta 1, e12. doi: 10.1002/imt2.12. PMID: 38868573 PMC10989754

[B49] Rousseau-Gueutin M. Belser C. Da Silva C. Richard G. Istace B. Cruaud C. . (2020). Long-read assembly of the Brassica napus reference genome Darmor-bzh. GigaScience 9, giaa137. doi: 10.1093/gigascience/giaa137. PMID: 33319912 PMC7736779

[B50] Saraiva A. L. R. F. Bhering A. S. do Carmo M. G. F. Andreote F. D. Dias A. C. F. Coelho I. S. (2020). Bacterial composition in brassica-cultivated soils with low and high severity of clubroot. J. Phytopathol. 168, 613–619. doi: 10.1111/jph.12941. PMID: 41940437

[B51] Shah N. Li Q. Xu Q. Liu J. Huang F. Zhan Z. X. . (2020). CRb and PbBa8.1 synergically increases resistant genes expression upon infection of Plasmodiophora brassicae in Brassica napus. Genes 11, 202. doi: 10.3390/genes11020202, PMID: 32079196 PMC7074261

[B52] Uroz S. Calvaruso C. Turpault M. P. Frey-Klett P. (2009). Mineral weathering by bacteria: ecology, actors and mechanisms. Cell 17, 378–387. doi: 10.1016/j.tim.2009.05.004. PMID: 19660952

[B53] Wang W. Qin L. Zhang W. Tang L. Zhang C. Dong X. . (2023). WeiTsing, a pericycle-expressed ion channel, safeguards the stele to confer clubroot resistance. Cell 186, 2656–2671. doi: 10.1016/j.cell.2023.05.023. PMID: 37295403

[B54] Wang Y. Y. Yang Z. Q. Yang Q. Y. Zhang C. Y. (2021). Genetic improvement and application of clubroot resistance in Brassica napus varieties. J. Huazhong Agric. Univ. 40, 1–5. doi: 10.1007/s11032-019-1056-6. PMID: 41940407

[B55] Wei X. Zhang Y. Zhao Y. Xie Z. Hossain M. R. Yang S. . (2021). Root transcriptome and metabolome profiling reveal key phytohormone-related genes and pathways involved clubroot resistance in Brassica rapa L. Front. Plant Sci. 12. doi: 10.3389/fpls.2021.759623. PMID: 34975941 PMC8715091

[B56] Wu C. Shi Y. Zhou Y. Xiong Q. Zhang C. Chen P. . (2025a). Current status of clubroot resistant rapeseed breeding and utilization in China and its main problems and countermeasures. Chin. J. Oil Crop Sci. 47, 513–525.

[B57] Wu Y. Zhao C. Zhang Y. Shen C. Zhang Y. Zhang X. . (2025b). Inactivation of β-1,3-glucan synthase-like 5 confers broad-spectrum resistance to Plasmodiophora brassicae pathotypes in cruciferous plants. Nat. Genet. 57, 2302–2312. doi: 10.1016/j.jviromet.2017.02.002. PMID: 40890362 PMC12425816

[B58] Xi D. D. Gao L. Miao L. M. Ge L. A. Zhang D. Y. Zhang Z. H. . (2023). Changes in diversity and composition of rhizosphere bacterial and fungal community between resistant and susceptible pakchoi under Plasmodiophora brassicae. Int. J. Mol. Sci. 24, 16779. doi: 10.3390/ijms242316779. PMID: 38069101 PMC10706474

[B59] Yu F. Q. Zhang Y. Wang J. H. Chen Q. L. Karium M. M. Gossen B. D. . (2022). Identification of two major QTLs in Brassica napus lines with introgressed clubroot resistance from turnip cultivar ECD01. Front. Plant Sci. 12. doi: 10.3389/fpls.2021.785989. PMID: 35095960 PMC8790046

[B60] Zhang Y. Cao G. Li X. Piao Z. (2023). Effects of exogenous ergothioneine on Brassica rapa clubroot development revealed by transcriptomic analysis. Int. J. Mol. Sci. 24, 6380. doi: 10.3390/ijms24076380. PMID: 37047350 PMC10094275

[B61] Zhang Y. L. Gan G. Y. Li Y. R. Li W. L. Jiang Y. Q. Wang P. . (2024). Exploring the temporal dynamics of a disease suppressive rhizo-microbiome in eggplants. iScience 27, 110319. doi: 10.1016/j.isci.2024.110319. PMID: 39055957 PMC11269921

[B62] Zhang Y. Jiang M. Ma J. Chen J. Kong L. Zhan Z. . (2025). Metabolomics and transcriptomics reveal the function of trigonelline and its synthesis gene BrNANMT in clubroot susceptibility of Brassica rapa. Plant Cell Environ. 15474, 1–14. doi: 10.1111/pce.15474. PMID: 40079515 PMC13551631

[B63] Zhao Y. Chen X. Cheng J. Xie J. Lin Y. Jiang D. . (2022). Application of Trichoderma Hz36 and Hk37 as biocontrol agents against clubroot caused by Plasmodiophora brassicae. J. Fungi 8, 777. doi: 10.4324/9781003303305-18. PMID: 35893144 PMC9331738

[B64] Zhou Q. Q. Galindo-González L. Manolii V. Hwang S. F. Strelkov S. E. (2020). Comparative transcriptome analysis of rutabaga (Brassica napus) cultivars indicates activation of salicylic acid and ethylene-mediated defenses in response to Plasmodiophora brassicae. Int. J. Mol. Sci. 21, 8381. doi: 10.3390/ijms21218381. PMID: 33171675 PMC7664628

[B65] Zhu M. He Y. Ren T. Liu H. Huang J. Jiang D. . (2020). Two new biocontrol agents against clubroot caused by Plasmodiophora brassicae. Front. Microbiol. 10:L1509. doi: 10.3389/fmicb.2019.03099. PMID: 32038545 PMC6986203

