# Supplementary figures and images for: Comparative analysis of the rhizosphere microbiome and transcriptome in clubroot-susceptible and resistant rapeseed (Brassica napus)
Source: Front Plant Sci. 2026 Apr 21;17:1729220. doi: 10.3389/fpls.2026.1729220 (PMC13139148; doi:10.3389/fpls.2026.1729220)

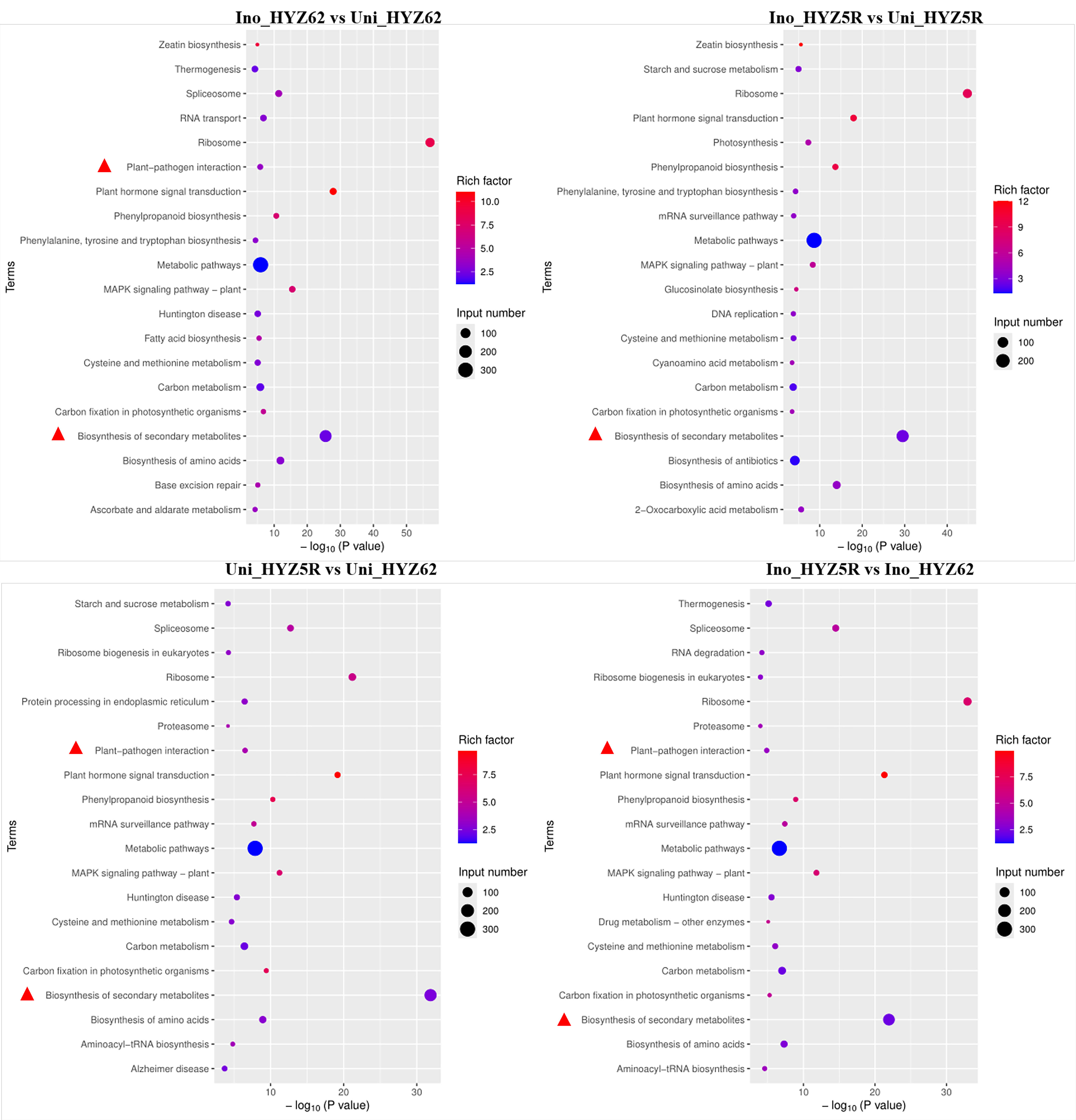

Supplement: Supplementary Figure S1 — KEGG pathway enrichment analysis of DEGs from four comparative groups. The four comparisons are: Ino_HYZ62 vs Uni_HYZ62, Ino_HYZ5R vs Uni_HYZ5R, Uni_HYZ5R vs Uni_HYZ62, Ino_HYZ5R vs Ino_HYZ62. The bubble plot displays the top significantly enriched KEGG pathways for each comparison. The size of the bubble represents the number of DEGs enriched in the pathway, and the color indicates the enrichment factor. Pathways marked with a red triangle were selected for detailed subsequent analysis. HYZ62 and HYZ5R were two rapeseed varieties (Supplementary Table S1). Plants in the Uninoculated (Uni) and Inoculated (Ino) treatments were treated with 2 mL of water and resting spore suspension (1 × 10^8^ resting spores/mL), respectively. [file Image1.tif]

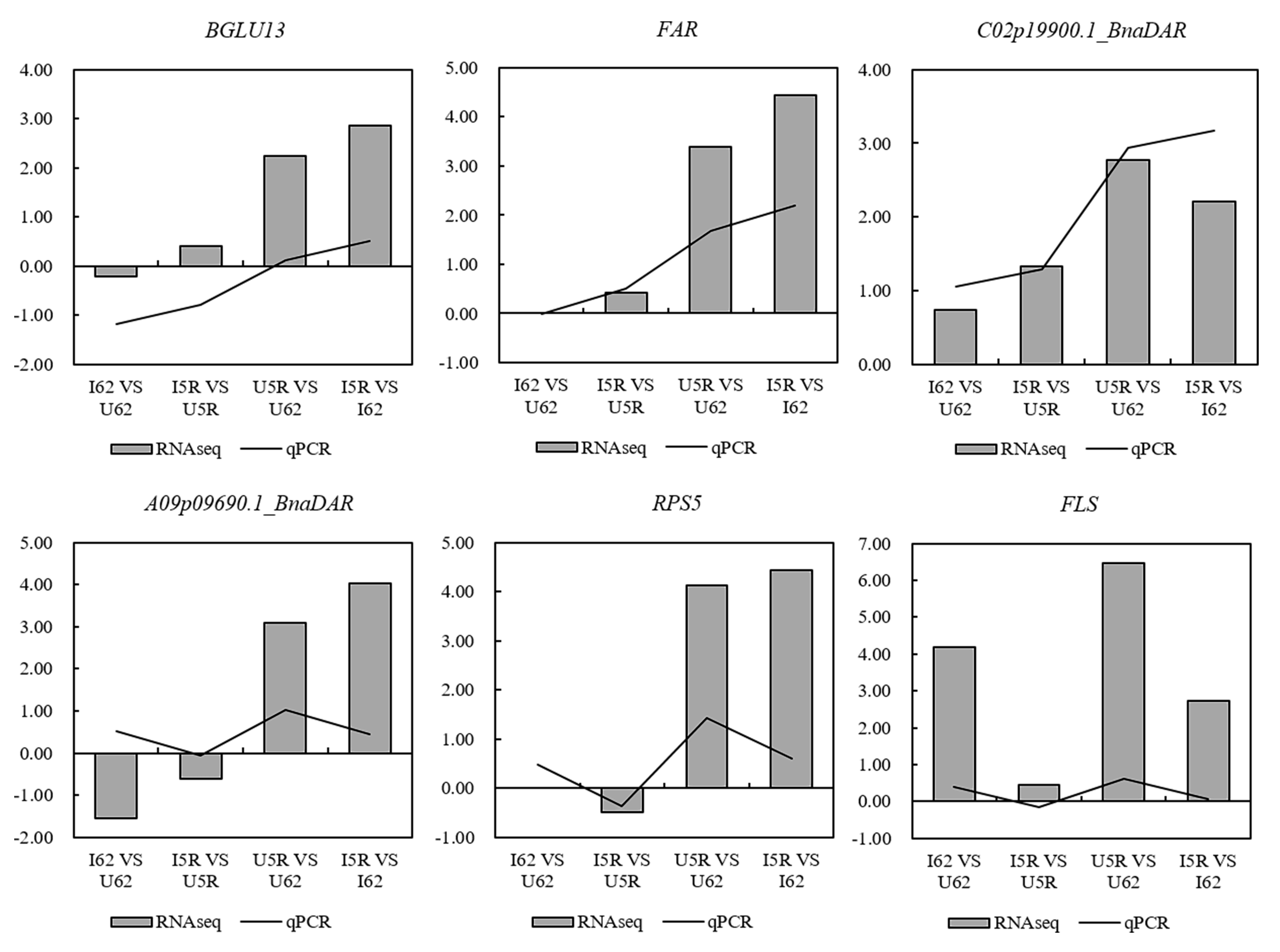

Supplement: Supplementary Figure S2 — Validation of RNA-seq data by qPCR for six selected genes. Bar and line graph showing the log2FC change of gene expression derived from the RNA-seq and qPCR, respectively. Gene expression levels in qPCR were normalized to BnTUB (B. napus tubulin beta chain, LOC111213844). Detailed gene information is available in Supplementary Table S2. The experimental treatments included: inoculated HYZ62 (I62), uninoculated HYZ62 (U62), inoculated HYZ5R (I5R), and uninoculated HYZ5R (U5R). HYZ62 and HYZ5R were two rapeseed varieties (Supplementary Table S1). Plants in the Uninoculated (Uni) and Inoculated (Ino) treatments were treated with 2 mL of water and resting spore suspension (1 × 10^8^ resting spores/mL), respectively. [file Image2.tif]

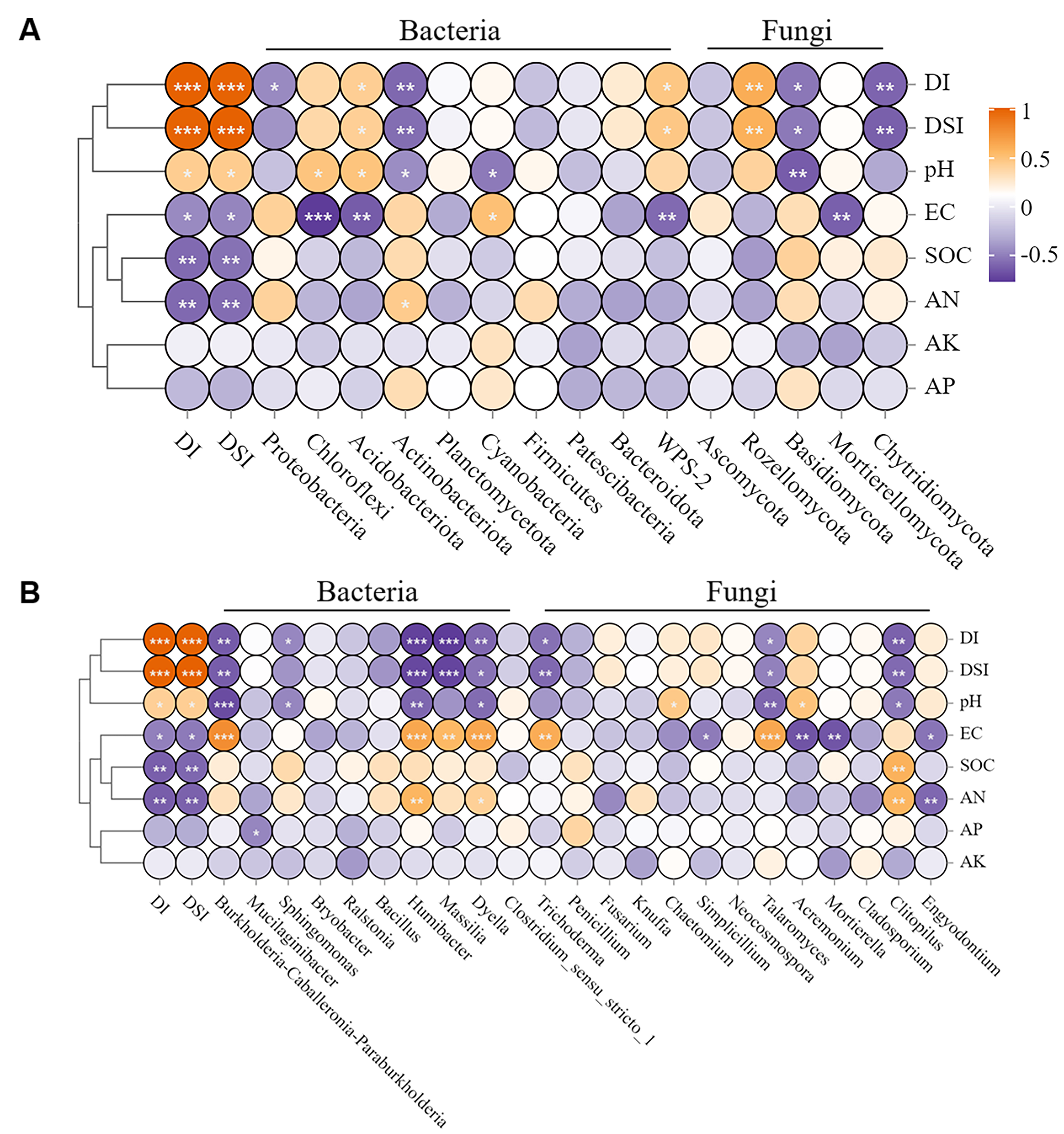

Supplement: Supplementary Figure S3 — Spearman correlation heatmap between rhizosphere microbial taxa, clubroot severity, and soil physicochemical properties. (A) Phylum-level and (B) genus-level bacterial and fungal taxa. The matrices display Spearman’s rank correlation coefficients (r) between microbial taxa and disease parameters (DI, disease incidence; DSI, disease index) or soil physicochemical properties (pH; EC, electrical conductivity; SOC, soil organic carbon; AN, alkali-hydrolysable nitrogen; AP, available phosphorus; AK, available potassium. Only taxa with a relative abundance > 1% in at least one treatment are shown. Asterisks indicate significant differences (*P < 0.05, **P < 0.01, ***P < 0.001). The dendrogram on the left represents hierarchical clustering of the disease and soil parameters based on their correlation patterns with the microbiota. The color scale indicates the correlation coefficient, with purple and orange representing negative and positive correlations, respectively.Table legends. [file Image3.tif]
