# Supplementary material for: Comparative analysis of the rhizosphere microbiome and transcriptome in clubroot-susceptible and resistant rapeseed (Brassica napus)
Source: Front Plant Sci. 2026 Apr 21;17:1729220. doi: 10.3389/fpls.2026.1729220 (PMC13139148; doi:10.3389/fpls.2026.1729220)
Supplement: Supplementary Table S1 — Names and sources of the six rapeseed varieties tested. [file Table1.docx]

Table S1 Names and sources of the six rapeseed varieties tested

| # | Name of the varieties | Abbreviation | Sources |
| --- | --- | --- | --- |
| 1 | Huayouza 62 | HYZ62 | Hubei Guoke High-tech Co., Ltd |
| 2 | Huashuang 5R | HS5R | Yida Agricultural Development Co., Ltd., Badong County, Hubei Province |
| 3 | Chaoji Meiguo Youwang | YW | Henan Zhuyou Seed Industry Co., Ltd |
| 4 | Fangyou 135R | FY135R | Sichuan Fangpai Seed industry Co., Ltd |
| 5 | Huayouza 160R | HYZ160R | Gucheng Shengguang Seed Industry Co., Ltd |
| 6 | Huayouza 5R | HYZ5R | Hubei Lizhong Seed Industry Technology Co., Ltd |
