# Supplementary material for: Comparative analysis of the rhizosphere microbiome and transcriptome in clubroot-susceptible and resistant rapeseed (Brassica napus)
Source: Front Plant Sci. 2026 Apr 21;17:1729220. doi: 10.3389/fpls.2026.1729220 (PMC13139148; doi:10.3389/fpls.2026.1729220)
Supplement: Supplementary Table S2 — The information on qPCR primers. [file Table2.docx]

Table S2 The information of qPCR primers

| Gene ID | Name | Description | Sequence（5’-3’） | |
| --- | --- | --- | --- | --- |
| *LOC111213844* (reference gene) | *BnTUB* | Tubulin beta chain | F | TTCGCACCTCTCACATCCC |
|  |  |  | R | AGTAGCGTCCGTGTCTTGG |
| *C04p58420.1_BnaDAR* | *BGLU13* | Beta glucosidase | F | GAGCCACTTGTGACCGGTAA |
|  |  |  | R | CGTTAGAGCAGGGGACATCC |
| *A05p37370.1_BnaDAR* | *FAR* | Alcohol-forming fatty acyl-CoA reductase | F | ATAAACACACGAGGGCCAGG |
|  |  |  | R | TCTTGCAGCATCAAGAGCCA |
| *C02p19900.1_BnaDAR* | / | / | F | CAGCGTCTTATCCGGCAGAT |
|  |  |  | R | CGTTGACTCTCCGTTGACCA |
| *A09p09690.1_BnaDAR* | / | / | F | ATGCCTTGCCCCACAATCTT |
|  |  |  | R | GATTGTGGGAGGCAAGCTCT |
| *C08p15270.1_BnaDAR* | *RPS5* | Resistance to *Pseudomonas syringae* 5 | F | ACGCTGCACCTATCTGCTTT |
|  |  |  | R | GACCAGTTCTTTACCCCCGG |
| *C02p62820.1_BnaDAR* | *FLS* | Flavonol synthase | F | TCTGGCCACCGTCAAGAATC |
|  |  |  | R | CTCAAGTTTTGGACACGGCG |
