# Supplementary material for: Comparative analysis of the rhizosphere microbiome and transcriptome in clubroot-susceptible and resistant rapeseed (Brassica napus)
Source: Front Plant Sci. 2026 Apr 21;17:1729220. doi: 10.3389/fpls.2026.1729220 (PMC13139148; doi:10.3389/fpls.2026.1729220)
Supplement: Supplementary Table S3 — Incidence and disease index of clubroot on six rapeseed varieties. HYZ62, HS5R, YW, FY135R, HYZ160R, HYZ5R were six rapeseed varieties (Supplementary Table S1). Values represent the mean of three biological replicates ± SE. Plants were treated with 2 mL resting spore suspension (1 × 10^8^ resting spores/mL). All data were collected at four weeks post-inoculation. Different lowercase letters indicate significant differences based on one-way ANOVA (P < 0.05, LSD test). [file Table3.docx]

Table S3 Incidence and disease index of clubroot on six rapeseed varieties

| # | Varieties | Disease incidence (%) | Disease index (DSI) |
| --- | --- | --- | --- |
| 1 | HYZ62 | 96.97 ± 3.03 a | 54.86 ± 2.50 a |
| 2 | HS5R | 93.33 ± 3.33 ab | 53.11 ± 2.85 a |
| 3 | YW | 85.61 ± 3.06 b | 57.77 ± 4.51 a |
| 4 | FY135R | 37.12 ± 2.44 c | 29.86 ± 3.87 b |
| 5 | HYZ160R | 31.56 ± 3.40 cd | 25.69 ± 0.69 bc |
| 6 | HYZ5R | 22.73 ± 2.27 d | 17.05 ± 2.08 c |
