# Supplementary material for: Comparative analysis of the rhizosphere microbiome and transcriptome in clubroot-susceptible and resistant rapeseed (Brassica napus)
Source: Front Plant Sci. 2026 Apr 21;17:1729220. doi: 10.3389/fpls.2026.1729220 (PMC13139148; doi:10.3389/fpls.2026.1729220)
Supplement: Supplementary Table S4 — Relative abundances of bacteria and fungi at the phylum level. Values represent the mean of six biological replicates; error bars represent SE. Asterisks indicate significant differences between Ino_HYZ5R and Ino_HYZ62, and between Uni_HYZ5R and Uni_HYZ62 (Student’s t-test *P < 0.05, **P < 0.01, ***P < 0.001). Bold text indicates a significant difference (Student’s t-test, P < 0.05) for the pairwise comparisons between Ino_HYZ5R and Uni_HYZ5R, and between Ino_HYZ62 and Uni_HYZ62. The experimental treatments included: inoculated HYZ62 (Ino_HYZ62), uninoculated HYZ62 (Uni_HYZ62), inoculated HYZ5R (Ino_HYZ5R), and uninoculated HYZ5R (Uni_HYZ5R). [file Table4.docx]

Table S4 Relative abundances of bacteria and fungi at the phylum level.

| Kingdom | Phylum | Relative abundance (%) | | | |
| --- | --- | --- | --- | --- | --- |
|  |  | Ino_HYZ5R | Ino_HYZ62 | Uni_HYZ5R | Uni_HYZ62 |
| Bacteria | Proteobacteria | 28.41 ± 4.93 | 23.92 ± 3.34 | 29.98 ± 2.06 | 26.21 ± 2.13* |
|  | Chloroflexi | **12.85 ± 1.04** | 14.47 ± 1.09* | 10.99 ± 1.55 | 13.92 ± 2.22* |
|  | Acidobacteriota | 10.14 ± 0.81 | 13.25 ± 0.76*** | 9.44 ± 1.13 | 12.31 ± 1.34** |
|  | Actinobacteriota | 8.61 ± 1.62 | 7.43 ± 1.03 | 10.42 ± 2.02 | 8.56 ± 0.71 |
|  | Planctomycetota | 7.33 ± 1.18 | 8.57 ± 1.14 | 7.45 ± 1.07 | 8.60 ± 0.96 |
|  | Cyanobacteria | 8.92 ± 6.11 | 6.08 ± 1.38 | 6.92 ± 2.18 | 4.44 ± 1.59* |
|  | Patescibacteria | **5.01 ± 0.53** | 6.32 ± 0.99* | 7.39 ± 1.98 | 5.73 ± 1.17 |
|  | Firmicutes | 7.24 ± 3.60 | **4.95 ± 1.26** | 5.55 ± 0.91 | 6.65 ± 0.99 |
|  | Bacteroidota | **3.23 ± 0.82** | 5.39 ± 0.80** | 4.50 ± 0.81 | 4.40 ± 1.34 |
|  | WPS-2 | 3.49 ± 0.22 | 4.57 ± 0.32*** | 3.46 ± 0.54 | 4.12 ± 0.70 |
| Fungi | Ascomycota | 79.03 ± 1.83 | 74.63 ± 1.61 | 82.38 ± 1.97 | 74.79 ± 2.64* |
|  | Rozellomycota | **9.43 ± 1.25** | 12.10 ± 1.12 | 4.56 ± 0.85 | 7.44 ± 1.77 |
|  | Basidiomycota | 1.40 ± 0.30 | 1.63 ± 0.58 | 2.77 ± 0.53 | 2.51 ± 0.63 |
|  | Mortierellomycota | 0.64 ± 0.14 | 2.96 ± 0.58** | 1.04 ± 0.18 | 3.65 ± 0.65** |
|  | Chytridiomycota | 0.46 ± 0.12 | **0.66 ± 0.16** | 1.84 ± 0.65 | 1.89 ± 0.44 |
