# Supplementary material for: Comparative analysis of the rhizosphere microbiome and transcriptome in clubroot-susceptible and resistant rapeseed (Brassica napus)
Source: Front Plant Sci. 2026 Apr 21;17:1729220. doi: 10.3389/fpls.2026.1729220 (PMC13139148; doi:10.3389/fpls.2026.1729220)
Supplement: Supplementary Table S5 — Relative abundances of bacteria and fungi at the genus level. Values represent the mean of six biological replicates; error bars represent SE. The experimental treatments included: inoculated HYZ62 (Ino_HYZ62), uninoculated HYZ62 (Uni_HYZ62), inoculated HYZ5R (Ino_HYZ5R), and uninoculated HYZ5R (Uni_HYZ5R). The significant differences between the two treatments were analyzed using the Student’s t-test. [file Table5.docx]

Table S5 Relative abundance of bacteria and fungi at the genus level

| Kingdom | Genus | Relative abundance (%) | | | | Uni_HYZ5R vs Uni_HYZ62 | Ino_HYZ5R vs Ino_HYZ62 | Ino_HYZ5R vs Uni_HYZ5R | Ino_HYZ62 vs Uni_HYZ62 |
| --- | --- | --- | --- | --- | --- | --- | --- | --- | --- |
|  |  | Ino_HYZ5R | Ino_HYZ62 | Uni_HYZ5R | Uni_HYZ62 |  |  |  |  |
| Bacteria | *Burkholderia-Caballeronia-Paraburkholderia* | 5.20 ± 0.21 | 2.77 ± 0.39 | 7.64 ± 0.42 | 3.97 ± 0.37 | ***P*=0.0000**  **+92.56%** | ***P*=0.0006**  **+87.61%** | ***P*=0.0009**  **-31.92%** | *P*=0.0512 |
|  | *Mucilaginibacter* | 2.03 ± 0.25 | 3.26 ± 0.20 | 3.13 ± 0.24 | 2.75 ± 0.11 | *P*=0.1793 | ***P*=0.0042**  **-37.67%** | ***P*=0.0126**  **-35.21%** | *P*=0.0523 |
|  | *Sphingomonas* | 2.24 ± 0.23 | 2.38 ± 0.08 | 3.03 ± 0.20 | 2.50 ± 0.08 | ***P*=0.0318**  **+21.23%** | *P*=0.5477 | ***P*=0.0296**  **-25.93%** | *P*=0.3418 |
|  | *Bryobacter* | 1.94 ± 0.09 | 2.40 ± 0.14 | 2.12 ± 0.13 | 2.47 ± 0.10 | *P*=0.0545 | ***P*=0.0267**  **-19.22%** | *P*=0.2833 | *P*=0.6689 |
|  | *Ralstonia* | 1.32 ± 0.32 | 1.75 ± 0.36 | 1.66 ± 0.33 | 1.59 ± 0.22 | *P*=0.8537 | *P*=0.3945 | *P*=0.4740 | *P*=0.7024 |
|  | *Bacillus* | 1.70 ± 0.12 | 1.56 ± 0.21 | 1.75 ± 0.14 | 2.24 ± 0.13 | ***P*=0.0281**  **-22.02%** | *P*=0.6016 | *P*=0.8153 | ***P*=0.0196**  **-30.28%** |
|  | *Humibacter* | 1.31 ± 0.20 | 0.62 ± 0.09 | 2.26 ± 0.31 | 0.99 ± 0.06 | ***P*=0.0027**  **+127.01%** | ***P*=0.0081**  **+111.94%** | ***P*=0.0394**  **-41.73%** | ***P*=0.0054**  **-37.58%** |
|  | *Massilia* | 0.77 ± 0.13 | 0.52 ± 0.04 | 1.54 ± 0.16 | 0.75 ± 0.06 | ***P*=0.0010**  **+104.21%** | *P*=0.0905 | ***P*=0.0058**  **-50.10%** | ***P*=0.0109**  **-30.60%** |
|  | *Dyella* | 0.84 ± 0.18 | 0.36 ± 0.06 | 1.34 ± 0.06 | 0.53 ± 0.04 | ***P*=0.0000**  **+152.19%** | ***P*=0.0255**  **+133.86%** | ***P*=0.0186**  **-37.70%** | ***P*=0.0340**  **-32.81%** |
|  | *Clostridium_sensu_stricto_1* | 1.06 ± 0.79 | 0.09 ± 0.02 | 0.11 ± 0.02 | 0.12 ± 0.01 | *P*=0.8108 | *P*=0.2106 | *P*=0.2186 | *P*=0.2862 |
| Fungi | *Trichoderma* | 19.29 ± 14.24 | 8.31 ± 1.23 | 29.56 ± 4.19 | 10.70 ± 1.50 | ***P*=0.0017**  **+176.40%** | *P*=0.0961 | *P*=0.1972 | *P*=0.2476 |
|  | *Penicillium* | 9.15 ± 1.77 | 7.42 ± 1.25 | 9.21 ± 1.96 | 10.39 ± 1.14 | *P*=0.6138 | *P*=0.4340 | *P*=0.9804 | *P*=0.1083 |
|  | *Fusarium* | 4.86 ± 0.85 | 7.20 ± 1.39 | 4.16 ± 0.37 | 4.64 ± 0.44 | *P*=0.4224 | *P*=0.2060 | *P*=0.4362 | *P*=0.1093 |
|  | *Knufia* | 3.20 ± 0.77 | 2.73 ± 0.15 | 2.95 ± 0.38 | 2.82 ± 0.33 | *P*=0.8092 | *P*=0.5226 | *P*=0.7592 | *P*=0.8036 |
|  | *Chaetomium* | 2.37 ± 0.47 | 2.50 ± 0.43 | 1.61 ± 0.77 | 2.34 ± 0.41 | *P*=0.4235 | *P*=0.8504 | *P*=0.4438 | *P*=0.7965 |
|  | *Simplicillium* | 0.81 ± 0.33 | 4.73 ± 0.61 | 1.30 ± 0.61 | 4.42 ± 1.44 | *P*=0.0741 | ***P*=0.0005**  **-82.90%** | *P*=0.5245 | *P*=0.8469 |
|  | *Neocosmospora* | 3.45 ± 1.04 | 1.65 ± 0.22 | 2.32 ± 0.61 | 1.23 ± 0.23 | *P*=0.1279 | *P*=0.0969 | *P*=0.3527 | 0.2164 |
|  | *Talaromyces* | 1.23 ± 0.35 | 1.58 ± 1.02 | 3.62 ± 2.00 | 1.00 ± 0.16 | *P*=0.2212 | *P*=0.7692 | *P*=0.3123 | *P*=0.5858 |
|  | *Acremonium* | 0.43 ± 0.23 | 2.61 ± 0.54 | 0.33 ± 0.13 | 2.68 ± 1.08 | *P*=0.0565 | ***P*=0.0075**  **-83.70%** | *P*=0.7059 | *P*=0.9547 |
|  | *Mortierella* | 0.64 ± 0.14 | 2.95 ± 0.58 | 1.04 ± 0.18 | 3.65 ± 1.60 | ***P*=0.0031**  **-71.61%** | ***P*=0.0066**  **-78.41%** | *P*=0.1243 | *P*=0.4420 |
|  | *Cladosporium* | 2.10 ± 0.88 | 2.32 ± 0.44 | 1.12 ± 0.37 | 2.92 ± 1.30 | *P*=0.2124 | *P*=0.8170 | *P*=0.3011 | *P*=0.6689 |
|  | *Clitopilus* | 0.15 ± 0.06 | 0.13 ± 0.08 | 1.45 ± 0.58 | 1.35 ± 0.62 | *P*=0.9119 | *P*=0.8346 | *P*=0.0744 | *P*=0.0769 |
|  | *Engyodontium* | 0.85 ± 0.29 | 1.47 ± 0.29 | 0.65 ± 0.13 | 1.33 ± 0.36 | *P*=0.1029 | *P*=0.1703 | *P*=0.5190 | *P*=0.7702 |
